# Asthma and obesity: endotoxin another insult to add to injury?

**DOI:** 10.1042/CS20210790

**Published:** 2021-12-17

**Authors:** Nikita Lad, Alice M. Murphy, Cristina Parenti, Carl P. Nelson, Neil C. Williams, Graham R. Sharpe, Philip G. McTernan

**Affiliations:** 1 Department of Biosciences, School of Science and Technology, Nottingham Trent University, Nottingham, NG11 8NS, U.K.; 2 SHAPE Research Centre, School of Science and Technology, Nottingham Trent University, Nottingham, NG11 8NS, U.K.

**Keywords:** Asthma, Gut microbiota, lipopolysaccharides, Obesity, Prebiotic, Probiotic

## Abstract

Low-grade inflammation is often an underlying cause of several chronic diseases such as asthma, obesity, cardiovascular disease, and type 2 diabetes mellitus (T2DM). Defining the mediators of such chronic low-grade inflammation often appears dependent on which disease is being investigated. However, downstream systemic inflammatory cytokine responses in these diseases often overlap, noting there is no doubt more than one factor at play to heighten the inflammatory response. Furthermore, it is increasingly believed that diet and an altered gut microbiota may play an important role in the pathology of such diverse diseases. More specifically, the inflammatory mediator endotoxin, which is a complex lipopolysaccharide (LPS) derived from the outer membrane cell wall of Gram-negative bacteria and is abundant within the gut microbiota, and may play a direct role alongside inhaled allergens in eliciting an inflammatory response in asthma. Endotoxin has immunogenic effects and is sufficiently microscopic to traverse the gut mucosa and enter the systemic circulation to act as a mediator of chronic low-grade inflammation in disease.

Whilst the role of endotoxin has been considered in conditions of obesity, cardiovascular disease and T2DM, endotoxin as an inflammatory trigger in asthma is less well understood. This review has sought to examine the current evidence for the role of endotoxin in asthma, and whether the gut microbiota could be a dietary target to improve disease management. This may expand our understanding of endotoxin as a mediator of further low-grade inflammatory diseases, and how endotoxin may represent yet another insult to add to injury.

## Why does asthma matter?

Asthma is an inflammatory disease of the airways affecting 262 million people worldwide [1], with a cost to the United States of $81.9 billion per year [2], and to Europe of €19.3 billion a year [3]. The United Kingdom contributes substantially to the European costs, with an annual charge of £4.9 billion, £1.1 billion of which is paid by the National Health Service (NHS) [3,4]. As such the financial burden for health economies is clear, but as asthma is often not considered a life-threatening disease in the main part, agents to relieve symptoms have been the main driver, with new therapies less forthcoming than other low-grade chronic inflammatory diseases in recent years. This may in part be due to less overall funding given for asthma research, as the National Institute of Health reported recently that asthma research received approximately $388 million in funding, compared with billions of dollars of funding for the areas of cardiovascular ($2.5 billion), diabetes ($1.2 billion) and obesity ($1.1 billion) research [5]. Despite the reduced comparable funding, asthma can be a deliberating, life-threatening, lifelong inflammatory disease with significant quality of life and health economy impact.

Asthma may arise as the human airway moves into a state of hypersensitivity, described as airway hyperresponsiveness (AHR) and occurs due to an increased production of inflammatory mediators including histamine and inflammatory cytokines, of which leucocytes (or white blood cells; WBCs) are a source. This AHR results in bronchoconstriction, increased mucus production and leads to symptoms including coughing, wheezing, shortness of breath and chest tightness. Several factors can increase the risk of developing asthma, such as, genetics and lifestyle, as well as various environmental triggers and allergens. Allergens can include particulate matter such as dust, dust mites, pollutants, smoking and pollen.

**Figure 1:**
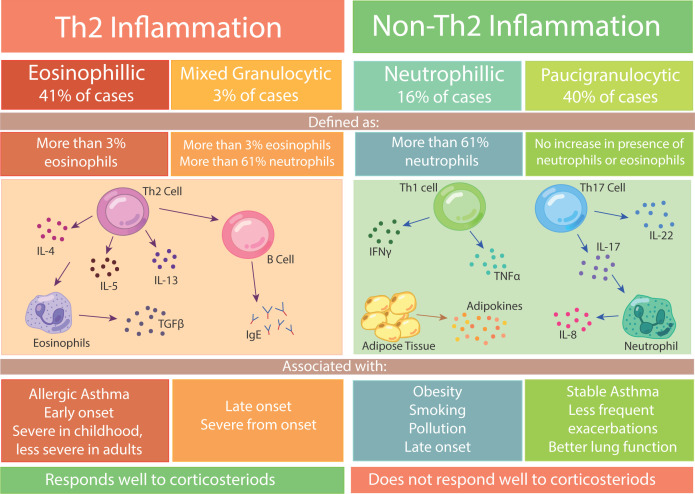
The phenotypes of severe asthma Inflammation in severe asthma can be divided into Th2 or Non-Th2. Th2 inflammation can be eosinophilic or mixed granulocytic, and is mainly associated with allergic asthma and early-onset asthma. Non-Th2 inflammation is either neutrophilic or paucigranulocytic, with the former being mostly associated with lifestyle factors such as obesity and smoking. Th2 inflammation responds well to corticosteroids, where as non-Th2 inflammation does not respond well to corticosteroids.

Asthma severity can range from mild through to moderate and severe. Severe asthma, defined as asthma which is uncontrolled or requires multiple therapies in order for it to become controlled [6], can be categorised into two inflammatory phenotypes, based on the inflammatory response present in the patient. The severe phenotypes are T-Helper cell 2 (Th2) mediated and non-Th2 mediated, which indicate whether or not Th2 cells are present as part of the inflammatory response. These severe phenotypes can be further categorised based on the immune cells that are present during an asthmatic episode (Figure 1).

Two of the main classes of immune cells that help characterise these severe phenotypes of asthma are eosinophils and neutrophils. Eosinophils have a bilobed nucleus and can initiate an allergic immune response, which leads to AHR and mucus overproduction in asthma. Neutrophils are the most abundant WBCs, they have a multilobed nucleus and are a type of phagocyte cell, able to engulf bacteria. In asthma, neutrophils contribute to chronic inflammation through the release of pro-inflammatory cytokines including, interleukin (IL)-8, IL-1β and tumour necrosis factor α (TNFα).

The Th2 phenotype can either be defined as eosinophilic asthma, in which there is an increase in eosinophils, or mixed granulocytic if there is a surge in both eosinophils and neutrophils (Figure 1). The non-Th2 phenotype is associated with neutrophilic asthma, which is dominated by neutrophils, or is paucigranulocytic, if there is no increase in either eosinophils or neutrophils. It has been established from prior studies that amongst subjects with asthma, 41% appear to be eosinophilic, 40% paucigranulocytic, 16% neutrophilic and 3% were mixed granulocytic [7]. Understanding the type of leucocyte that is activated during an exacerbation is crucial in order to provide the correct asthma treatments to patients, as 56% of patients fall under the non-Th2 phenotype, which tends to be more poorly controlled and does not respond well to treatments [8,9]. The non-Th2 phenotype of severe asthma, which arises due to the neutrophilic immune response, is also associated with corticosteroid resistance [10,11], corticosteroids being used as a common therapy for most asthma cases.

In cases where asthma can be controlled by corticosteroids and leukotriene modifiers, the medication mitigates the inflammatory response and reverses bronchoconstriction. Specifically, the medication reduces inflammation by suppressing the activity of leucocytes such as eosinophils, which subsequently leads to the reduction in inflammatory cytokines. Other medicines such as short- and long-acting β agonists can relax the airway smooth muscle to widen the airway and provide noted relief. However, persistent use of these medications can cause common side effects (1 in 100 people) including muscle cramps, increased heart rate, headaches and feeling unstable, noting that β agonists are not always well tolerated by patients [12–14]. Since current therapies act predominantly to reduce symptoms, there is a clear need to develop new therapies to ameliorate inflammation at source, rather than targeting the arising symptoms.

## Role of obesity in asthma

Clinical obesity (BMI over 30 kg/m^2^) appears to be a critical risk factor for developing asthma in both children and adults [15,16]. In U.S.A., 60% of adults with severe asthma are obese [16]. Furthermore, studies continue to re-affirm that asthma prevalence amongst adults is higher in obese adults (11.1%) than in lean adults (7.1%), with the highest prevalence being in obese women (14.6%) compared with obese men (7.1%) [17]. In addition, obese women appear more likely to have more frequent exacerbations [18]. Combined with the known elevated risk of chronic inflammatory diseases in non-Caucasian adults, the odds ratio (OR) of developing asthma rises if subjects are obese, altered with varying ethnicities: African American (OR: 2.9) and Hispanic males (OR: 2.7) [19] and Indian (OR: 1.92) and Chinese women (OR: 2.1) [20,21].

Obesity may increase the risk of asthma, as with weight gain adipose tissue itself has an increased response to inflammatory cells and stimuli, through activated leucocytes and an increase in the release of adipokines via other mediators. Indirectly, it has also been suggested that excess abdominal visceral fat accumulation in obesity can exacerbate asthma due do the excess fat causing increased pressure on the diaphragm, which in turn constricts the lungs [22,23].

The impact of obesity to heighten the risk of asthma is also observed in babies born to obese mothers where babies are at a higher risk of developing asthma, with an OR of 1.31–1.34 [24,25]. This risk is furthered, if, weight gain in the child continues in infancy, where the risk of developing asthma is sustained with a similar OR of 1.30 [26–28]. The prevalence of asthma in children increases proportionally with changes in BMI [29], with children who are obese exhibiting a two-fold increased risk in developing asthma compared with non-obese children [30].

People with asthma and obesity also tend to develop more severe asthma due to the presence of neutrophilic inflammation and the increased inflammatory response adipose tissue poses compared with lean individuals with asthma. As such, as the volume of adipose tissue increases, the immune cell composition within the adipose tissue shifts from a Th2 response to a Th1/17 response [31] with an increased presence of neutrophilic inflammation [32]. In individuals with asthma and obesity, inflammation appears to be predominately driven by the Th1/17 cellular response [33–35], leading to less symptomatic control by corticosteroid medication, as they typically target the Th2 response, meaning less well-controlled exacerbations and a more severe asthmatic response [32,36]. In addition to inhaled allergens heightening this immune response and inflammation in asthma, obesity may further exacerbate this inflammatory response due to the increased level of endotoxin noted in obese patients [37].

## Molecular links amongst asthma, diet and obesity

Beyond several known causes that directly affect lung function such as inhalation of irritants including dust mites, pollen and pollutants, other *in vivo* mediators of asthma appear to play their part. Similar to individuals with asthma, an underlying systemic inflammation profile is also observed in people during weight gain. These pro-inflammatory cytokines, released from adipose tissue and referred to as adipokines [38], activate the innate immune response in adipocytes leading to further inflammation [39–41]. As the volume of adipose tissue in the body rises, as in obesity, there is an associated pro-inflammatory profile of adipokines produced. These adipokines can have a range of systemic cellular and damaging functional organ effects including on lung tissue (Table 1). As such, adipokines provides an insight into how obesity can influence asthma and other chronic diseases.

**Table 1:** List of the effect of adipokines in adipose tissue and airway cells

Adipokine	Effect in adipose tissue	Effect in airway cells	References
Leptin	Increases lipolysis Promotes adipogenesis Causes the release of pro-inflammatory cytokines including IL-6 and TNFα	Causes bronchodilation Increases production of chemokines and cytokines - Eotaxin, MCP-1, IL-8, IL-6 and CXCL10	[42,43]
Adiponectin	Increases glucose uptake in fat cells Enhances adipogenesis and lipid storage	Increases release of anti-inflammatory cytokine IL-10 Decreases release of pro-inflammatory IL-6 and TNFα	[44]
IL-6	Increases leptin secretion and lipolysis Suppresses satiety signals, therefore increasing hunger	Promotes ciliogenesis	[45,46]
Resistin	Inhibits adiponectin secretion and induces lipolysis Activates innate immune response Regulates expression of PAI-1	Up-regulates mucin production	[39,47–49]
TNFα	Causes mitochondrial dysfunction Alters adipokine production Induces lipolysis Impairs insulin signalling	Induces apoptosis in cells infected by *Legionella pneumophila*	[50–54]
Angiotensin	Activates Ca^2+^ signalling pathways Promotes adipocyte browning	Angiotensin I converted into angiotensin II in lungs	[55,56]
Visfatin	Involved in brown adipocyte thermogenesis and can decrease UCP-1 expression at high concentrations	Increases mucin production via activation of NF-κB pathway	[57,58]
MCP-1	Causes insulin resistance and recruits macrophages	Up-regulates mucin production through CCR2 receptor	[59,60]
TGF-β1	Regulates adipocyte browning	Induces PAI-1 expression in airway epithelial cells	[61,62]
PAI-1	Causes inflammation	Causes AHR, inflammation and remodelling	[63,64]
IL-8	Causes insulin resistance via inhibition of Akt phosphorylation	Increases Ca^2+^ release from airway smooth muscles cells, leads to constriction of airways	[65,66]
IL-10	Prevents adipocyte differentiation and lipid accumulation	Reduces airway inflammation and hyperresponsiveness	[67,68]
IL-17α	Induced expression of TNFα, IL-6, IL-1β, leptin, and glucose transporter 4	Causes bronchoconstriction and AHR	[69,70]
IL-1β	Inhibits insulin signalling and glucose transport Increases lipolysis Increases inflammation	Involved in airway cell migration	[71,72]

Effect of adipokines on adipose tissue and airway cells which may lead to widespread systemic effects in disease. In adipose tissue, this may include increased inflammation, altered lipid and glucose homoeostasis and regulation of adipocyte browning. In airway cells, adipokines can control the inflammatory response, AHR, bronchoconstriction, bronchodilation and mucin production. In both cell types, these effects can lead to an exacerbation or relief from inflammatory diseases.

Abbreviations: CCR2, C–C chemokine receptor 2; CXCL10, C–X–C motif chemokine ligand 10; MCP-1, monocyte chemoattractant protein 1; NF-κB, nuclear factor κ-light-chain-enhancer of activated B cell; PAI-1, plasminogen activator inhibitor 1; TGF-β1, transforming growth factor β 1; UCP-1, uncoupling protein 1.

Beyond the considered normal function of these adipokines in routine homoeostasis such as satiety, blood pressure and glucose regulation, these factors have functions which can impact the health of lung tissue. This occurs when the balance of pro- and anti-inflammatory factors, often mainly derived from adipose tissue, is shifted towards a pro-inflammatory state. From prior studies it has been observed that obese subjects with asthma have more circulating leptin and a reduction in the anti-inflammatory adipokine, adiponectin, than lean people with asthma [34,73–75]. This appears important as in patients with asthma, leptin induces inflammation in lung fibroblasts by enhancing the production of further pro-inflammatory chemokines and cytokines [42], which appears somewhat suppressed when a patient experiences leptin resistance. In contrast, adiponectin can have an anti-inflammatory effect in airway cells by promoting the release of the anti-inflammatory cytokine IL-10 and the inhibition of pro-inflammatory cytokines IL-6 and TNFα [76]. However due to the shift in leptin and adiponectin levels in obese patients with asthma, the adipokines can mediate more inflammation and AHR.

Coupled with weight gain, increasing the systemic release of pro-inflammatory factors is also known to be influenced by diet, which may indirectly lead to asthma exacerbations. It is understood that insulin resistance and glucose intolerance are associated with severity in asthma cases [29,77–79]. It appears that circulating glucose indirectly causes inflammation, as hyperglycaemia induces oxidative stress which in turn increases inflammation [80,81]. This inflammatory insult caused by hyperglycaemia can persist in the long term, even after glucose levels are better controlled, due to the ‘metabolic memory’ that has been observed in cells such as adipocytes [82]. As such, glucose homoeostasis appears to represent an important factor in the development of severe asthma and poor lung function, which may occur as excess glucose increases levels of systemic inflammation.

Furthermore, compared with healthy controls, individuals with asthma and metabolic syndrome in the form of a combination of obesity, type 2 diabetes mellitus (T2DM), hypertension and/or hyperlipaemia are observed to experience a 10% decrease in lung function, whilst subjects with asthma alone have a 6% reduction in function [83]. This 4% difference may not appear much between the two conditions, however, combined with the other inflammatory tissue responses, this could enhance severity of the asthma response markedly. Whilst it is apparent that other chronic inflammatory conditions can exacerbate asthma, there has been some suggestion that obesity alone may not always be sufficient to drive heightened AHR, with a particular study by Karampatakis and co-workers highlighting that only subjects with obesity and impaired glucose control and/or insulin resistance may drive AHR [78].

However it is understood that other factors such as cholesterol, triglycerides and elevated free fatty acids (FFAs) can also drive inflammation [84,85], with sustained raised systemic levels postprandially and where ectopic fat deposition challenges arise in obesity [86,87]. These lipids can induce inflammation in adipose tissue by activating the innate immune system through toll-like receptors (TLRs) [82,88].

The TLRs themselves form part of a repertoire of germline-encoded pattern recognition receptors (PRRs) to sense inflammatory factors. The major PRRs include TLRs, Nod-like receptors (NLRs), retinoic acid-inducible gene 1 (RIG-I)-like receptors and C-type lectins. TLRs and NLRs can be activated by a variety of dietary factors in response to obesity-induced metabolic stress. This stress can arise from nutrient excess, inducing modification of the gut microbiota and increased gut permeability which may trigger an influx of various microbiota-derived, pathogen-associated molecular patterns into the circulation that activate their corresponding PRRs in many tissues. Both TLR2 and TLR4 have been shown to sense FFAs; in addition, ceramides, heat shock proteins and modified LDLs can also activate TLR4. Following activation of TLR2 and TLR4, they can signal through MyD88-dependent and MyD88-independent pathways to activate the nuclear factor κ-light-chain-enhancer of activated B cell (NF-κB) and MAPK pathways to inhibit insulin signalling through insulin receptor substrate (IRS) serine phosphorylation and to induce the transcription of pro-inflammatory cytokines, such as TNF-α, IL-6, pro-IL-1 and pro-IL-18. Nutrients such as long-chain saturated fatty acids, ceramides, modified LDL, high glucose levels and cholesterol crystals have been shown to activate NLR-protein 3, possibly through induction of reactive oxygen species (ROS). NLRP3 then assembles with the adaptor protein, apoptosis-associated speck-like protein containing a caspase-recruitment domain (ASC) and caspase-1 into a multiprotein complex called the inflammasome, which cleaves the inactive precursors of pro-IL-1 and pro-IL-18 to the active forms of IL-1 and IL-18 [89–94]. Furthermore, FFAs have been shown to directly activate the inflammasome in airway smooth muscle cells, where long-chain FAs activate the receptor free fatty acid receptor 1 (FFAR1), leading to bronchoconstriction and enhanced asthma symptoms [86].

Studies in asthma also suggest that high serum triglycerides and low-density lipoprotein cholesterol (LDL-C) are also associated with cases of asthma amongst obese children and adolescents [29,34,95]. Serum triglycerides and LDL-C were also identified to be associated with reduced lung function in adults [96–98].

Obesity and diet can contribute to an increased inflammatory response, which can lead to cellular damage and dysfunction in lung tissue. However, it appears that diet may not be the sole contributing factor arising from the gut to mediate inflammation in asthma, as the microbiota may also have a lead role to play.

## Endotoxin-induced inflammation

**Figure 2:**
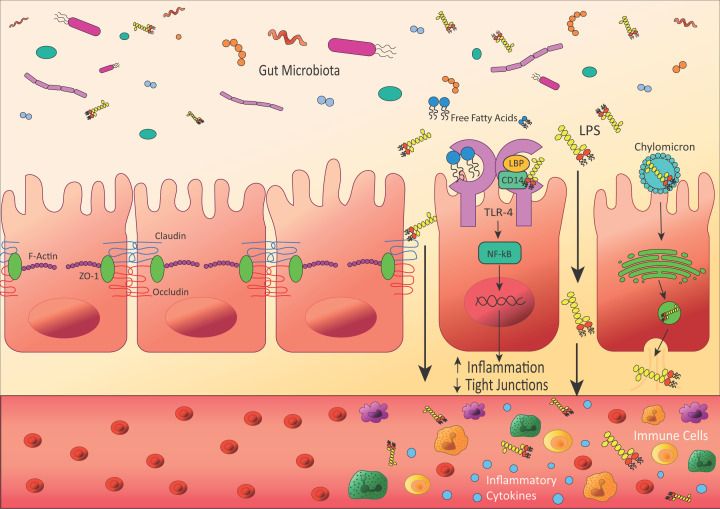
Leaky gut barrier leads to systemic inflammation In healthy patients, the gut epithelia form a barrier, connected by tight junction proteins including claudins, occludins and ZO-1, to prevent molecules in the gut lumen from crossing into the blood. However, in diseases including asthma and obesity, tight junctions can become weak, allowing molecules of endotoxin (LPS) to cross into the circulatory system, which can cause an immune response and inflammation. This can occur through several mechanisms; firstly, LPS can bind to TLR-4, which activates the NF-κB signalling pathway and increases the expression of inflammatory cytokines. Secondly, when LPS binds to TLR-4 it can also lead to a signalling cascade that decreases the expression of tight junction proteins, weakening the gut barrier and allowing more LPS to cross. Finally, LPS can be transported into gut cells by chylomicrons, which usually transport fat to the liver and adipose tissues. LPS is taken up by the chylomicrons, they enter the cell and are packaged by the Golgi apparatus, before exiting the cells and into the circulatory system via vesicles.

Endotoxin, referred to as lipopolysaccharide (LPS), is a molecule that consists of lipid A, which is anchored into the outer membrane of Gram-negative bacteria and is responsible for the toxic activity, connected to a polysaccharide which consists of an oligosaccharide core and a distal O-antigen [37]. It is the catalytic activity of the Lipid A component of LPS, which is similar in structure to FFAs, that is able to activate the innate immune cascade via TLRs and mediates an inflammatory response. Whilst endotoxin predominantly remains in the gut, endotoxin is also able to traverse the gut mucosa via several distinct mechanisms (Figure 2). These mechanisms may arise through endotoxin being attached to chylomicrons, a lipoprotein that usually transports FFAs [114], or dysfunctional and increased permeability of the gut barrier, which allows increased levels of bacterial extracellular vesicles containing endotoxin to enter the circulation [115]. Several health disorders associated with low-grade inflammation have been shown to involve increased intestinal permeability, including inflammatory bowel disease, coeliac disease, obesity, T2DM and asthma [116–121].

Besides the functional changes to enhance endotoxin entry into the circulation, diet can also enhance the level of endotoxin in the bloodstream. The consumption of a high saturated fat meal resulted in increased circulated endotoxin, noted in patients with chronic low-grade inflammation [122], as well as healthy adults [123,124] and in mouse models [118]. It is considered that chylomicron release, increases in high fat diets, leads to increased postprandial endotoxin levels in obese individuals after a high-fat meal [125]. Levels of the associated endotoxin protein CD14 have also been shown to increase during digestion, which coincides with a postprandial peak in IL-6 [126] and a wider inflammatory response.

**Figure 3:**
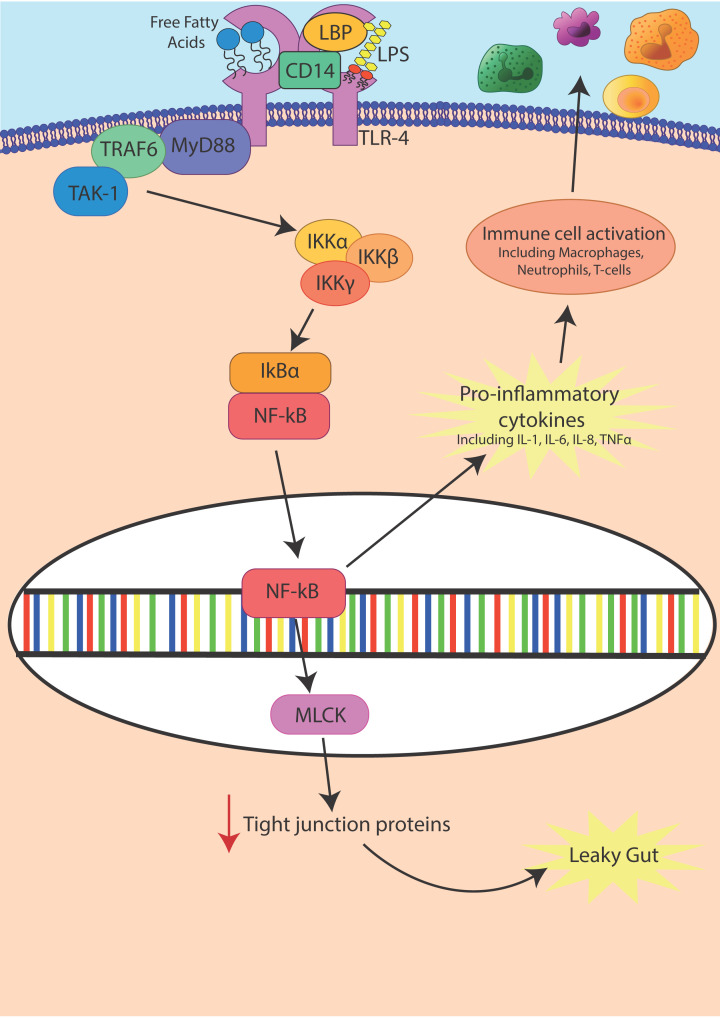
Endotoxin causes inflammation and leaky gut through activation of NF-κB pathways Endotoxin (LPS) causes inflammation through the activation of NF-κB via TLR-4. LPS is detected by LPS-binding protein (LBP). LBP presents the LPS to cluster of differentiation 14 (CD14). CD14 then allows LPS to bind to TLR-4 and activate the NF-κB signalling pathway leading to increased release of inflammatory cytokines. NF-κB also increases transcription of MLCK, which decreases the transcription of tight junction proteins, causing the gut barrier to weaken and become leaky.

Increased circulating endotoxin can then lead to raised inflammation in adipose tissue due to the release of pro-inflammatory cytokines [127]. Endotoxin may mediate an inflammatory response via TLR-4 through the activation of the NF-κB pathway (Figure 3). Expression of TLR-4 can be increased by endotoxin itself [128]. The pro-inflammatory cytokines released then go on to activate an innate immune response through the recruitment of macrophages, neutrophils and T cells [129]. In addition, endotoxin can mediate functional tight junction permeability changes through this same NF-κB pathway activating the Myosin Light Chain Kinase (*MLCK*) gene, which then decreases tight junction protein expression to promote a leaky gut [130–132].

## Endotoxin in asthma

Endotoxin is known to be associated with both neutrophilic and eosinophilic airway inflammation, AHR and corticosteroid resistance in asthma, [133–138] enhancing systemic pro-inflammatory cytokines [115,139]. In addition, studies have observed that endotoxin is able to increase Th2 cytokine IL-13 secretion and reduce responsiveness to corticosteroid treatment [140]. Furthermore, it has been suggested that endotoxin is able to cause asthma phenotypes to shift from eosinophilic to neutrophilic [141], by promoting differentiation of CD4^+^ cells into Th17 cells rather than Th2 cells [142]. This shift would therefore lead to corticosteroid-resistant, poorly controlled asthma and an enhanced severity of the condition.

The level of endotoxin in the environment *per se* can vary but generally remains below 10 EU/m^3^ in urban and rural areas [143–145]. When inhaled into the lungs, it can affect a patient’s severity of asthma. Factors including farming and air pollution can increase the ambient endotoxin levels and have been linked to respiratory issues [146–148]. It had been considered that early life exposure to endotoxin may be protective against developing allergic asthma by suppressing Th2 inflammatory mechanisms [149,150]. However, recent studies appear to show that infants with a recurrent wheeze have raised levels of endotoxin in their sputum [151]. Furthermore, the apparent protective action of endotoxin has also been noted to be lost beyond infancy, where endotoxin exposure becomes associated with the onset of asthma in teenage years [152] and adulthood [153].

It has also been shown that the cellular response to endotoxin is dependent on the structure of the molecule itself [154]. Lipid A is typically hexa- or penta-acylated, with the hexa-acylated form able to cause a 100-fold higher immune response [155]. Exposure to the different types of lipid A may also depend on geographical location or climate [156]. It has been observed that people living in urban areas appear more likely to be exposed to the penta-acylated lipid A, contained within *Bacteroidetes* and *Prevotella* bacterial species and as a result they are less likely to have asthma [157,158].

Changes in intestinal permeability have also been reported in asthma patients [116,117,120,159], however a causal link amongst gut-derived endotoxin, intestinal permeability and respiratory inflammation is yet to be fully explored. Ultimately, current evidence suggests that both intestinal and systemic inflammation are derived from altered microbiota patterns, where endotoxin may lead to enhance systemic cell pro-inflammatory activation and tissue inflammation [127,160,161].

## Impact of the microbiota on health

From our current knowledge, due to the eminent complexity of the human microbiota, it is considered that everyone has their own unique microbial signature, heavily influenced by genetics, environmental and lifestyle factors. This begins prior to birth and the moment of birth, where the altered exposure to microbes coupled with a reduction in *Bifidobacterium* colonisation leads to an increased risk of atopic and inflammation borne diseases in infants born via c-section delivery [99]. This *Bifidobacterium* colonisation can be further influenced by diet, with a plant-based diet associated with increased beneficial strains of *Bifidobacterium* and *Lactobacillus* whilst an animal-based diet is more associated with an increase in *Bacteroides* and *Biophilia* [100,101]. Furthermore, beyond diet, ageing itself has also been shown to shift microbiota diversity in key bacterial genus including an increase in *Bacteroides* and *Enterobacteriaceae* and a decrease in *Bifidobacterium* [102].

Whilst the gut microbiota may be considered the grand central in our whole-body microflora, there are distinct microbial communities across the human body that are also important [103]. Even within tissues there is diversity [103], as well as in different regions of the gut, where despite the adjoined locality such as in the duodenum and jejunum the levels of the type of strains vary [104]. Beyond the diversity, the interplay of these communities could impact our health as well as the response patients have to an asthmatic episode. It is considered that an optimal healthy bacterial microbiota community should be diverse and well balanced. A reduction in diversity and a bacterial microbiota imbalance, referred to as dysbiosis, can lead to an overgrowth of Gram-negative bacteria leading ultimately to the increased release of endotoxin. Dysbiosis, *per se* is known to drive an inflammatory response in the host and is considered a key factor in the development of chronic inflammatory bowel disease, obesity, T2DM, cardiovascular disease and asthma [105–113].

## Role of the airway and lung microbiota in asthma

There is emerging evidence that the composition of the airway microbiota plays an active role in the severity of bronchial hyperresponsiveness [162] and inflammatory phenotype [163,164] in patients with asthma. It is reported that patients with asthma tend to have more Gram-negative species of bacteria in their airway microbiota compared with those without asthma [109,165]. Furthermore, there is a specific increase in abundance of the Gram-negative genus *Moraxella* in the nasal [166] and airway microbiota [151,167,168] which is also associated with an increased risk of exacerbation. While genes associated with endotoxin biosynthesis are also observed as being raised in the nasal microbiota of young adults with asthma compared with those without asthma [109]. There is also a specific increase in the Gram-negative phylum *Proteobacteria* noted in the airway microbiota of patients with asthma [165,167,169] with expression of Th17-related genes correlated with this phylum [170].

Although both the eosinophilic and neutrophilic phenotypes of asthma show an increase in Gram-negative strains compared with healthy people, the neutrophilic phenotype was shown to have less bacterial diversity and more pathogenic and opportunistic strains in the airways [163]. There is also an increased bacterial load in the neutrophilic phenotype, so these associations are amplified when compared with the eosinophilic phenotype [167]. The increase in endotoxin-producing Gram-negative bacterial strains in the airway has been associated with corticosteroid resistance in severe asthma, due to high activation of the NF-κB pathway via TLR-4 activation [133]. Taken together, this suggests that there is an increased potential for airway-derived endotoxin to enter the circulation and induce inflammation due to an increase in Gram-negative strains observed in patients with asthma.

## Altered gut microbiota in people with asthma

The gut microbiota in chronic low-grade inflammatory diseases is associated with a reduced bacterial diversity, leading to a shift towards more Gram-negative bacterial strains and therefore more bioavailability of endotoxin, which can mediate the exacerbation of these conditions [171–174]. Although asthma is a disease characterised by inflammation within the airways exacerbated by inhaled allergens and individualised triggers; there is evidence to suggest a role of the gut microbiota in respiratory conditions. The association between the gut microbiota and the airways is termed the gut–lung axis [175]. Current studies suggests that early bacterial colonisation (and its diversity) of the gut influences whether an infant develops asthma [176–181]. Children with asthma have been shown to have a reduction in probiotic strains *Bifidobacterium* and *Lactobacilli*, with an increase in harmful strains including *Escherichia coli, Helicobacter pylori, Streptococcus* and *Staphylococcus*, which appears to result in increased inflammation [182]. Reduction in gut bacterial diversity through antibiotic use in pregnancy or early childhood also contributes to this increased risk [183,184]. Currently, there are limited studies exploring the impact of the gut microbiota on asthma risk in adults, although a recent pilot study has observed a correlation between opportunistic bacterial strains including *Prevotella* and poor lung function in adults with asthma [185].

Murine studies mirror the importance of early life exposure to diverse microbial environments, as mice raised in a germ-free environment which is free of any bacteria, had an increased risk of developing asthma symptoms and inflammation via Th2 activation resulting in increased IL-4 and IL-5 production [186]. Several murine studies using vancomycin, to alter the gut microbiota, have shown subsequently in recolonisation that more Gram-negative bacteria colonise the gut, and that these mice are at a higher risk of developing asthma [187–189]. These studies clearly describe a link between Gram-negative bacteria and the risk of developing asthma with a rise in the availability of endotoxin, and as such a potential for endotoxin-induced inflammation to be a mediator of asthma. These human and rodent studies also highlight how antibiotic use may increase the risk of developing asthma. As such, the ability to enhance gut microbiota diversity through diet may be an important method to reduce systemic endotoxin, reduce the arising inflammation and improve the condition of patients with asthma. As such, modifying the composition of the microbiota by encouraging the growth of beneficial bacteria strains could lead to a reduction in inflammation caused by the harmful bacteria strains. A simple and relatively cheap way of improving the diversity of the microbiota is through diet and may therefore offer a strategy, in part, to reduce inflammation and the arising asthma symptoms.

## Dietary use of pro/prebiotics as a treatment for asthma

Natural therapies that alter the gut microbiota such as pre- and probiotics are becoming more widely accessible and gaining popularity and media attention. Probiotics involve the delivery of live strains of beneficial bacteria to the gut in order to improve the health of the host [190]. They can be consumed through yoghurt-based drinks, tablets or in fermented foods such as pickled vegetables, kimchi and soy. A prebiotic is a non-digestible carbohydrate which is utilised by bacteria in the gut and alters the composition of the gut microbiota by encouraging the growth of beneficial strains such as *Bifidobacteria* and *Lactobacilli* [191]. The main types of prebiotics that are commonly used are inulins, a group of polysaccharides, and galactooligosaccharides (GOS), an oligosaccharide linked with galactose, both of which are types of plant-derived fibres.

When prebiotics are metabolised by bacteria, the metabolites produced may exert beneficial effects on the host and are termed postbiotics [192,193]. The main metabolites are short-chain fatty acids (SCFAs), which are classified as fatty acids with fewer than 6 carbon atoms, with acetate (C2), propionate (C3) and butyrate (C4) being the most abundant [194]. The amount of SCFA produced by bacteria varies depending on the prebiotic used, with GOS leading to the highest rate of production [195]. Studies have indicated that SCFAs have an anti-inflammatory effect [196–198], so prebiotics are therefore thought to be beneficial by increasing the levels of SCFAs produced by bacteria.

SCFAs activate free fatty acid receptor 2 (FFAR2) and FFAR3 (also referred to as GPR43 and GPR41 respectively). Both receptors are expressed in leucocytes, endothelial cells and airway smooth muscle and epithelial cells, whilst FFAR3 is also expressed in adipose tissue [199,200]. Expression of FFAR2 and FFAR3 in asthma patients appears to increase within 4 h of consuming a high-fibre meal [201]. FFAR3 has varied effects in different cell types, causing vasodilation in vascular smooth muscle cells [202]. Interestingly, it has been documented that patients with asthma have a reduced number of total SCFAs compared with healthy individuals, which is thought to arise due to a decrease in the metabolic activity of SCFA-producing bacteria [203]. Notably, a decrease in SCFA-producing *Veillonella* in a matter of months following birth has been associated with development of atopic wheeze later in childhood [177,178]. Infants with reduced levels of faecal SCFAs were also more likely to develop asthma and other atopic diseases later in life [204]. These findings are also confirmed by studies using pregnant mice, showing that reducing SCFA-producing bacteria with vancomycin leads to offspring with severe asthma symptoms [189]. Therefore, increasing SCFAs could be a potential novel therapy for asthma, either through diversifying bacterial species, or reducing endotoxin-induced inflammation, mitigating the inflammatory response observed in subjects with asthma.

Further studies also suggest that SCFAs can increase the expression of tight junction proteins including ZO-1, claudins and occludin, therefore enhancing the intestinal barrier [205–209]. In addition, SCFAs have also been shown to inhibit damage caused by endotoxin in the gut [208,210].

Although studies on the use of prebiotics to reduce asthma are limited, prior studies do suggest they may have beneficial effects. In human interventional trials using inulin and bimuno-galactoligosaccharide (B-GOS) in patients who experienced asthma or exercise-induced asthma respectively, both prebiotics were observed to reduce both inflammatory markers and AHR in participants [211,212]. Larger scale studies are needed before detailed conclusions can be made, however these studies along with several murine studies [213–215] highlight the potential of the bifidogenic effect to ease the symptoms of asthma.

Probiotics may provide a further option for mitigating bacterial dysbiosis in individuals suffering from chronic low-grade inflammation and atopic diseases, where bacterial diversity and levels of beneficial bacterial strains are relatively low. Probiotic intervention trials are complex and fraught with confounders, however studies appear to show that they can reduce inflammation in participants with T2DM [216] as well as in healthy individuals [217], although this appears for now to be the case over longer intervention periods rather than acute studies [218,219]. Furthermore, the specific use of *Lactobacillus GG* administered to pregnant women, and then to their children for 6 months, demonstrated that the children had a lower risk of developing atopic diseases including asthma [220]. Follow-up studies confirmed that the same children were still at a lower risk of atopic disease 7 years after the original study [221,222], although not all such studies have found similar findings [223,224]. The potential challenge with the use of probiotics is that even if the bacteria reach the gut, if they have inadequate food sources such as dietary fibre, which could be due to the host’s poor diet, the bacteria do not colonise sufficiently to exert their beneficial effect. This suggests that there needs to be some form of dietary intervention as well as the probiotic supplement for optimal results. Out of these studies to date, there is evidence to suggest *Bifidobacterium* and *Lactobacillus* are particularly able to reduce endotoxin-induced inflammation and increase tight junction protein expression, therefore improving intestinal permeability [225–227].

Studies have also sought to administer both pre- and probiotics together, known as synbiotics, to enhance health outcomes in asthma. Dietary interventions using a mixture of inulin and probiotics has been shown to reduce airway inflammation in participants with asthma within 4 h of consumption [201]. Animal studies also reported similar findings, with the synbiotic effect reducing both eosinophil and neutrophil cell counts, inflammation [228] and improving intestinal permeability [229]. However, conflicting reports have been noted in humans, where pre- and probiotics administered together had no synbiotic effect, even though when given separately they improved intestinal permeability [230]. This may be due to a number of confounders including competition between probiotics and established bacteria for nutrients, or the participants dietary habits being suboptimal for probiotic growth, so a more controlled diet may be required in future studies.

Insight into systemic endotoxin specifically has shown the levels to be reduced after either pre- or probiotic supplementation, however these studies have been limited to healthy participants [216,231] and patients with chronic metabolic diseases including obesity and T2DM [232–237]. Whilst studies in asthma have not yet considered the therapeutic reduction in endotoxin as a method to reduce symptoms or health outcomes in such patients. Therefore, future asthma studies including endotoxin as a measurement may give more insight into the role of endotoxin as an instigator of inflammation and how dietary intervention could reduce this inflammation.

## Conclusion

Endotoxin may represent an ever-present allergen for individuals with asthma, through both inhaling endotoxin that resides ambiently in the environment, as well as endotoxin derived from the airway and gut microbiota communities. The response to endotoxin at all levels appears to mount an innate immune inflammatory response that is exacerbated by comorbidities such as obesity. It is also apparent that the environment and diet affects the systemic availability and pathogenicity of endotoxin in asthma, which can also be mitigated by the type of environment, diet and dietary components that modulate the gut microbiota in humans. This suggest that endotoxin represents more than a dust mite in its potential action on individuals with asthma. Intervening with dietary therapeutics, such as pre- or probiotics, to disrupt the endotoxin-induced systemic inflammation may provide an effective route to reduce the severity of asthma. Ultimately, studies to date suggest that endotoxin may operate as an instigator of low-level chronic inflammation in asthma, and as such present an opportunity to target this allergen directly in individuals with asthma, rather than the arising symptoms which can be less easy to control.

## Abbreviations

AHR: airway hyperresponsiveness
BMI: Body mass index
FFA: free fatty acid
FFAR: free fatty acid receptor
GOS: galactoligosaccharide
IL: interleukin
LDL-C: low-density lipoprotein cholesterol
LPS: lipopolysaccharide
NF-κB: nuclear factor κ-light-chain-enhancer of activated B cell
NLR: Nod-like receptor
OR: odds ratio
PRR: pattern recognition receptor
SCFA: short-chain fatty acid
T2DM: type 2 diabetes mellitus
Th: T helper cell
TLR: Toll-like receptor
TNFα: tumour necrosis factor α
WBC: white blood cell
ZO-1: Zonula occludens-1

## References

[1] World Health Organisation. Asthma. https://www.who.int/news-room/fact-sheets/detail/asthma

[2] Nurmagambetov T., Kuwahara R. and Garbe P. (2018) The economic burden of asthma in the United States, 2008-2013. Ann. Am. Thorac. Soc. 15, 348–356 10.1513/AnnalsATS.201703-259OC 29323930

[3] Nunes C., Pereira A.M. and Morais-Almeida M. (2017) Asthma costs and social impact https://pmc/articles/PMC5219738/?report=abstract 10.1186/s40733-016-0029-3 28078100 PMC5219738

[4] Mukherjee M., Stoddart A., Gupta R.P., Nwaru B.I., Farr A., Heaven M. et al. (2016) The epidemiology, healthcare and societal burden and costs of asthma in the UK and its member nations: Analyses of standalone and linked national databases. BMC Med. 14, 113, http://bmcmedicine.biomedcentral.com/articles/10.1186/s12916-016-0657-8 10.1186/s12916-016-0657-8 27568881 PMC5002970

[5] National Institute of Health (2021) Estimates of Funding for Various Research, Condition, and Disease Categories (RCDC). https://report.nih.gov/funding/categorical-spending#/2021;

[6] Chung K.F., Wenzel S.E., Brozek J.L., Bush A., Castro M., Sterk P.J. et al. (2014) International ERS/ATS guidelines on definition, evaluation and treatment of severe asthma. Eur. Respir. J. 43, 343–373 10.1183/09031936.00202013 24337046

[7] Schleich F.N., Manise M., Sele J., Henket M., Seidel L. and Louis R. (2013) Distribution of sputum cellular phenotype in a large asthma cohort: predicting factors for eosinophilic vs neutrophilic inflammation. BMC Pulm. Med. 13, 11, https://pmc/articles/PMC3657295/?report=abstract 10.1186/1471-2466-13-11 23442497 PMC3657295

[8] Esteban-Gorgojo I., Antolín-Amérigo D., Domínguez-Ortega J. and Quirce S. (2018) Non-eosinophilic asthma: current perspectives. J. Asthma Allergy 11, 267–281 10.2147/JAA.S153097 30464537 PMC6211579

[9] Tliba O. and Panettieri R.A. (2019) Paucigranulocytic asthma: uncoupling of airway obstruction from inflammation. J. Allergy Clin. Immunol. 143, 1287–1294 10.1016/j.jaci.2018.06.008 29928921 PMC6301131

[10] Ray A. and Kolls J.K. (2017) Neutrophilic inflammation in asthma and association with disease severity. Trends Immunol. 38, 942–954, https://pmc/articles/PMC5711587/?report=abstract 10.1016/j.it.2017.07.003 28784414 PMC5711587

[11] Wadhwa R., Dua K., Adcock I.M., Horvat J.C., Kim R.Y. and Hansbro P.M. (2019) Cellular mechanisms underlying steroid-resistant asthma. Eur. Respir. Rev. 28, 190096 10.1183/16000617.0096-2019 31636089 PMC9488801

[12] Cates C.J. and Cates M.J. (2012) Regular treatment with formoterol for chronic asthma: serious adverse events. Cochrane Database Syst. Rev. 2012(4) CD006923, 10.1002/14651858.CD006923.pub3 PMC4017186 22513944

[13] Nelson H.S., Weiss S.T., Bleecker E.K., Yancey S.W. and Dorinsky P.M. (2006) The salmeterol multicenter asthma research trial: a comparison of usual pharmacotherapy for asthma or usual pharmacotherapy plus salmeterol. In Chest, pp. 15–26, American College of Chest Physicians 10.1378/chest.129.1.15 16424409

[14] Cates C.J., Wieland L.S., Oleszczuk M. and Kew K.M. (2014) Safety of regular formoterol or salmeterol in adults with asthma: an overview of Cochrane reviews. Cochrane Database Syst. Rev. 2014, CD010314 10.1002/14651858.CD010314.pub2 PMC7087438 24504983

[15] Forno E., Han Y.Y., Libman I.M., Muzumdar R.H. and Celedón J.C. (2018) Adiposity and asthma in a nationwide study of children and adults in the United States. Ann. Am. Thorac. Soc. 15, 322–330 10.1513/AnnalsATS.201709-723OC 29144884 PMC5880523

[16] Peters U., Dixon A.E. and Forno E. (2018) Obesity and asthma. J. Allergy Clin. Immunol. 141, 1169–1179 10.1016/j.jaci.2018.02.004 29627041 PMC5973542

[17] Akinbami L.J. and Fryar C.D. (2016) Current asthma prevalence by weight status among adults: United States, 2001-2014. NCHS Data Brief. 239, 1–8 27019018

[18] To M., Hitani A., Kono Y., Honda N., Kano I., Haruki K. et al. (2018) Obesity-associated severe asthma in an adult Japanese population. Respir. Investig. 56, 440–447 10.1016/j.resinv.2018.07.003 30100132

[19] Kim S. and Camargo C.A. (2003) Sex-race differences in the relationship between obesity and asthma: The behavioral risk factor surveillance system, 2000. Ann. Epidemiol. 13, 666–673 10.1016/S1047-2797(03)00054-1 14599730

[20] Celedón J.C., Palmer L.J., Litonjua A.A., Weiss S.T., Wang B., Fang Z. et al. (2001) Body mass index and asthma in adults in families of subjects with asthma in Anqing, China. Am. J. Respir. Crit. Care Med. 164, 1835–1840 10.1164/ajrccm.164.10.2105033 11734432

[21] Mishra V. (2004) Effect of obesity on asthma among adult Indian women. Int. J. Obes. 28, 1048–1058, www.nature.com/ijo 10.1038/sj.ijo.0802700 15197412

[22] Dixon A.E. and Peters U. (2018) The effect of obesity on lung function. Expert Rev. Respir. Med. 12, 755–767, https://pmc/articles/PMC6311385/?report=abstract 10.1080/17476348.2018.1506331 30056777 PMC6311385

[23] Yang M.S., Choi S., Choi Y. and Jin K.N. (2018) Association between airway parameters and abdominal fat measured via computed tomography in asthmatic patients. Allergy Asthma Immunol. Res. 10, 503–515, https://pmc/articles/PMC6082818/?report=abstract 10.4168/aair.2018.10.5.503 30088370 PMC6082818

[24] Forno E., Young O.M., Kumar R., Simhan H. and Celedón J.C. (2014) Maternal obesity in pregnancy, gestational weight gain, and risk of childhood asthma. Pediatrics 134, e535–46 10.1542/peds.2014-0439 25049351 PMC4187236

[25] Dumas O., Varraso R., Gillman M.W., Field A.E. and Camargo C.A. (2016) Longitudinal study of maternal body mass index, gestational weight gain, and offspring asthma. Allergy 71, 1295–1304 10.1111/all.12876 26969855 PMC4975656

[26] Popovic M., Pizzi C., Rusconi F., Galassi C., Gagliardi L., de Marco L. et al. (2016) Infant weight trajectories and early childhood wheezing: The NINFEA birth cohort study. Thorax 71, 1091–1096 10.1136/thoraxjnl-2015-208208 27369356

[27] Byberg K.K., Mikalsen I.B., Eide G.E., Forman M.R., Júlíusson P.B. and Øymar K. (2018) The associations between weight-related anthropometrics during childhood and lung function in late childhood: a retrospective cohort study. BMC Pulm. Med. 18, 10, https://bmcpulmmed.biomedcentral.com/articles/10.1186/s12890-017-0567-3 10.1186/s12890-017-0567-3 29351745 PMC5775530

[28] Casas M., den Dekker H.T., Kruithof C.J., Reiss I.K., Vrijheid M., Sunyer J. et al. (2018) The effect of early growth patterns and lung function on the development of childhood asthma: a population based study. Thorax 73, 1137–1145 10.1136/thoraxjnl-2017-211216 30064992

[29] Cottrell L., Neal W.A., Ice C., Perez M.K. and Piedimonte G. (2011) Metabolic abnormalities in children with asthma. Am. J. Respir. Crit. Care Med. 183, 441–448, https://pmc/articles/PMC3056222/?report=abstract 10.1164/rccm.201004-0603OC 20851922 PMC3056222

[30] Chen Y.C., Dong G.H., Lin K.C. and Lee Y.L. (2013) Gender difference of childhood overweight and obesity in predicting the risk of incident asthma: a systematic review and meta-analysis. Obes. Rev. 14, 222–231 10.1111/j.1467-789X.2012.01055.x 23145849

[31] Karczewski J., Śledzińska E., Baturo A., Jończyk I., Maleszko A., Samborski P. et al. (2018) Obesity and inflammation. Eur. Cytokine Netw. 29, 83–94 10.1684/ecn.2018.0415 30547890

[32] Scott H.A., Gibson P.G., Garg M.L. and Wood L.G. (2011) Airway inflammation is augmented by obesity and fatty acids in asthma. Eur. Respir. J. 38, 594–602, https://erj.ersjournals.com/content/38/3/594 10.1183/09031936.00139810 21310876

[33] Kim H.Y., Lee H.J., Chang Y.J., Pichavant M., Shore S.A., Fitzgerald K.A. et al. (2014) Interleukin-17-producing innate lymphoid cells and the NLRP3 inflammasome facilitate obesity-associated airway hyperreactivity. Nat. Med. 20, 54–61 10.1038/nm.3423 24336249 PMC3912313

[34] Rastogi D., Fraser S., Oh J., Huber A.M., Schulman Y., Bhagtani R.H. et al. (2015) Inflammation, metabolic dysregulation, and pulmonary function among obese urban adolescents with asthma. Am. J. Respir. Crit. Care Med. 191, 149–160 10.1164/rccm.201409-1587OC 25457349 PMC4347436

[35] Everaere L., Ait-Yahia S., Molendi-Coste O., Vorng H., Quemener S., LeVu P. et al. (2016) Innate lymphoid cells contribute to allergic airway disease exacerbation by obesity. J. Allergy Clin. Immunol. 138, 1309.e11–1318.e11 10.1016/j.jaci.2016.03.019 27177781

[36] de Jesus J.P.V., Lima-Matos A.S., Almeida P.C.A., Lima V.B., de Mello L.M., Souza-Machado A. et al. (2018) Obesity and asthma: Clinical and laboratory characterization of a common combination. J. Bras. De Pneumol. 44, 207–212 10.1590/s1806-37562017000000034 PMC6188692 30043887

[37] Piya M.K., Harte A.L. and McTernan P.G. (2013) Metabolic endotoxaemia: is it more than just a gut feeling? Curr. Opin. Lipidol. 24, 78–85 10.1097/MOL.0b013e32835b4431 23298961

[38] Taylor E.B. (2021) The complex role of adipokines in obesity, inflammation, and autoimmunity. Clin. Sci. 135, 731–752 10.1042/CS20200895 PMC7969664 33729498

[39] Kusminski C.M., da Silva N.F., Creely S.J., Fisher F.M., Harte A.L., Baker A.R. et al. (2007) The in vitro effects of resistin on the innate immune signaling pathway in isolated human subcutaneous adipocytes. J. Clin. Endocrinol. Metab. 92, 270–276 10.1210/jc.2006-1151 17062773

[40] Conde J., Scotece M., Gómez R., López V., Gómez-Reino J.J., Lago F. et al. (2011) Adipokines: biofactors from white adipose tissue. A complex hub among inflammation, metabolism, and immunity. Biofactors 37, 413–420 10.1002/biof.185 22038756

[41] Mancuso P. (2016) The role of adipokines in chronic inflammation. Immuno Targets Ther. 5, 47–56, https://pmc/articles/PMC4970637/ 10.2147/ITT.S73223 27529061 PMC4970637

[42] Watanabe K., Suzukawa M., Arakawa S., Kobayashi K., Igarashi S., Tashimo H. et al. (2019) Leptin enhances cytokine/chemokine production by normal lung fibroblasts by binding to leptin receptor. Allergol. Int. 68, S3–S8 10.1016/j.alit.2019.04.002 31029506

[43] Palhinha L., Liechocki S., Hottz E.D., da Silva Pereira J.A., de Almeida C.J. et al. (2019) Leptin induces proadipogenic and proinflammatory signaling in adipocytes. Front. Endocrinol. 10, 841, https://www.frontiersin.org/article/10.3389/fendo.2019.00841/full 10.3389/fendo.2019.00841 31920961 PMC6923660

[44] Stern J.H., Rutkowski J.M. and Scherer P.E. (2016) Cell metabolism review adiponectin, leptin, and fatty acids in the maintenance of metabolic homeostasis through adipose tissue crosstalk. Cell Metab. 23, 770–784 10.1016/j.cmet.2016.04.011 27166942 PMC4864949

[45] Tadokoro T., Wang Y., Barak L.S., Bai Y., Randell S.H. and Hogan B.L.M. (2014) IL-6/STAT3 promotes regeneration of airway ciliated cells from basal stem cells. Proc. Natl. Acad. Sci. U.S.A. 111, 3641–3649 10.1073/pnas.1409781111 PMC4156689 25136113

[46] Wueest S. and Konrad D. (2018) The role of adipocyte-specific IL-6-type cytokine signaling in FFA and leptin release. Adipocyte 7, 226–228 10.1080/21623945.2018.1493901 30001663 PMC6224188

[47] Chen N., Zhou L., Zhang Z., Xu J., Wan Z. and Qin L. (2014) Resistin induces lipolysis and suppresses adiponectin secretion in cultured human visceral adipose tissue. Regul. Pept. 194-195, 49–54 10.1016/j.regpep.2014.10.001 25454366

[48] Ikeda Y., Tsuchiya H., Hama S., Kajimoto K. and Kogure K. (2014) Resistin regulates the expression of plasminogen activator inhibitor-1 in 3T3-L1 adipocytes. Biochem. Biophys. Res. Commun. 448, 129–133 10.1016/j.bbrc.2014.03.076 24667608

[49] Kwak S., Kim Y.D., Na H.G., Bae C.H., Song S.Y. and Choi Y.S. (2018) Resistin upregulates MUC5AC/B mucin gene expression in human airway epithelial cells. Biochem. Biophys. Res. Commun. 499, 655–661 10.1016/j.bbrc.2018.03.206 29604272

[50] Souza S.C., Palmer H.J., Kang Y.H., Yamamoto M.T., Muliro K.v., Paulson K.E. et al. (2003) TNF-α induction of lipolysis is mediated through activation of the extracellular signal related kinase pathway in 3T3-L1 adipocytes. J. Cell. Biochem. 89, 1077–1086 10.1002/jcb.10565 12898507

[51] Chen X.H., Zhao Y.P., Xue M., Ji C.B., Gao C.L., Zhu J.G. et al. (2010) TNF-α induces mitochondrial dysfunction in 3T3-L1 adipocytes. Mol. Cell. Endocrinol. 328, 63–69 10.1016/j.mce.2010.07.005 20667497

[52] Kawamoto Y., Morinaga Y., Kimura Y., Kaku N., Kosai K., Uno N. et al. (2017) TNF-α inhibits the growth of Legionella pneumophila in airway epithelial cells by inducing apoptosis. J. Infect. Chemother. 23, 51–55 10.1016/j.jiac.2016.09.010 27865699

[53] Peraldi P., Hotamisligil G.S., Buurman W.A., White M.F. and Spiegelman B.M. (1996) Tumor necrosis factor (TNF)-β inhibits insulin signaling through stimulation of the p55 TNF receptor and activation of sphingomyelinase. J. Biol. Chem. 271, 13018–13022 10.1074/jbc.271.22.13018 8662983

[54] Cawthorn W.P. and Sethi J.K. (2008) TNF-α and adipocyte biology. FEBS Lett. 582, 117–131, https://pmc/articles/PMC4304634/ 10.1016/j.febslet.2007.11.051 18037376 PMC4304634

[55] Dolgacheva L.P., Turovskaya M.V., Dynnik V.V., Zinchenko V.P., Goncharov N.V., Davletov B. et al. (2016) Angiotensin II activates different calcium signaling pathways in adipocytes. Arch. Biochem. Biophys. 593, 38–49 10.1016/j.abb.2016.02.001 26850364

[56] Than A., Xu S., Li R., Leow M.S., Sun L. and Chen P. (2017) Angiotensin type 2 receptor activation promotes browning of white adipose tissue and brown adipogenesis. Signal Transduct. Target. Ther. 2, 17022 10.1038/sigtrans.2017.22 29263921 PMC5661636

[57] Song S.Y., Jung E.C., Bae C.H., Choi Y.S. and Kim Y.D. (2014) Visfatin induces MUC8 and MUC5B expression via p38 MAPK/ROS/NF-κB in human airway epithelial cells. J. Biomed. Sci. 21, 49, 10.1186/1423-0127-21-49 24885580 PMC4041053

[58] Dimitriadis G.K., Adya R., Tan B.K., Jones T.A., Menon V.S., Ramanjaneya M. et al. (2019) Effects of visfatin on brown adipose tissue energy regulation using T37i cells. Cytokine 113, 248–255 10.1016/j.cyto.2018.07.013 30060995

[59] Dahlman I., Kaaman M., Olsson T., Tan G.D., Bickerton A.S.T., Wåhlén K. et al. (2005) A unique role of monocyte chemoattractant protein 1 among chemokines in adipose tissue of obese subjects. J. Clin. Endocrinol. Metab. 90, 5834–5840, https://academic.oup.com/jcem/article/90/10/5834/2839685 10.1210/jc.2005-0369 16091493

[60] Monzon M.E., Forteza R.M. and Casalino-Matsuda S.M. (2011) MCP-1/CCR2B-dependent loop upregulates MUC5AC and MUC5B in human airway epithelium. Am. J. Physiol. Lung Cell. Mol. Physiol. 300, 204–215 10.1152/ajplung.00292.2010 21097527 PMC3043814

[61] Cho S.H., Lee S.H., Kato A., Takabayashi T., Kulka M., Shin S.C. et al. (2015) Cross-talk between human mast cells and bronchial epithelial cells in plasminogen activator inhibitor-1 production via transforming growth factor-β1. Am. J. Respir. Cell Mol. Biol. 52, 88–95 10.1165/rcmb.2013-0399OC 24987792 PMC4370249

[62] Wankhade U.D., Lee J.H., Dagur P.K., Yadav H., Shen M., Chen W. et al. (2018) TGF-β receptor 1 regulates progenitors that promote browning of white fat. Mol. Metab. 16, 160–171, https://pmc/articles/PMC6158128/?report=abstract 10.1016/j.molmet.2018.07.008 30100246 PMC6158128

[63] Jo A., Lee S.H., Kim D.Y., Hong S.J., Teng M.N., Kolliputi N. et al. (2018) Mast cell-derived plasminogen activator inhibitor type 1 promotes airway inflammation and remodeling in a murine model of asthma. J. Allergy Clin. Immunol. 142, 294.e5–297.e5, https://www.ncbi.nlm.nih.gov/pmc/articles/PMC6046190/ 10.1016/j.jaci.2018.01.040 29477725 PMC6046190

[64] Liu Y., Wang L., Luo M., Chen N., Deng X., He J. et al. (2019) Inhibition of PAI-1 attenuates perirenal fat inflammation and the associated nephropathy in high-fat diet-induced obese mice. Am. J. Physiol. Endocrinol. Metab. 316, E260–E267 10.1152/ajpendo.00387.2018 30532990

[65] Govindaraju V., Michoud M.C., Ferraro P., Arkinson J., Safka K., Valderrama-Carvajal H. et al. (2008) The effects of interleukin-8 on airway smooth muscle contraction in cystic fibrosis. Respir. Res. 9, 76, http://respiratory-research.biomedcentral.com/articles/10.1186/1465-9921-9-76 10.1186/1465-9921-9-76 19046427 PMC2633308

[66] Kobashi C., Asamizu S., Ishiki M., Iwata M., Usui I., Yamazaki K. et al. (2009) Inhibitory effect of IL-8 on insulin action in human adipocytes via MAP kinase pathway. J. Inflamm. 6, 25, https://pmc/articles/PMC2746203/?report=abstract 10.1186/1476-9255-6-25 PMC2746203 19709445

[67] Branchett W.J. and Lloyd C.M. (2019) Regulatory cytokine function in the respiratory tract. Mucosal Immunol. 12, 589–600 10.1038/s41385-019-0158-0 30874596 PMC7051906

[68] Kim Y.H. and Pyo S. (2019) Interleukin-10 suppresses adipogenesis via Wnt5a signaling pathway in 3T3-L1 preadipocytes. Biochem. Biophys. Res. Commun. 509, 877–885 10.1016/j.bbrc.2019.01.033 30642634

[69] Kudo M., Melton A.C., Chen C., Engler M.B., Huang K.E., Ren X. et al. (2012) IL-17A produced by αβ T cells drives airway hyper-responsiveness in mice and enhances mouse and human airway smooth muscle contraction. Nat. Med. 18, 547–554, https://pmc/articles/PMC3321096/?report=abstract 10.1038/nm.2684 22388091 PMC3321096

[70] Qu Y., Zhang Q., Ma S., Liu S., Chen Z., Mo Z. et al. (2016) Interleukin-17A differentially induces inflammatory and metabolic gene expression in the adipose tissues of lean and obese mice. Int. J. Mol. Sci. 17, 522 https://pmc/articles/PMC4848978/?report=abstract 10.3390/ijms17040522 27070576 PMC4848978

[71] White S.R., Fischer B.M., Marroquin B.A. and Stern R. (2008) Interleukin-1β mediates human airway epithelial cell migration via NF-κB. Am. J. Physiol. Lung Cell. Mol. Physiol. 295, 10.1152/ajplung.00065.2008 PMC2604797 18849440

[72] Bing C. (2015) Is interleukin-1β a culprit in macrophage-adipocyte crosstalk in obesity? Adipocyte 4, 149–152, https://pmc/articles/PMC4496963/?report=abstract 10.4161/21623945.2014.979661 26167419 PMC4496963

[73] Sideleva O., Suratt B.T., Black K.E., Tharp W.G., Pratley R.E., Forgione P. et al. (2012) Obesity and asthma: an inflammatory disease of adipose tissue not the airway. Am. J. Respir. Crit. Care Med. 186, 598–605 10.1164/rccm.201203-0573OC 22837379 PMC3480522

[74] Nigro E., Daniele A., Scudiero O., Monaco M., Roviezzo F., D’Agostino B. et al. (2015) Adiponectin in asthma: implications for phenotyping. Curr. Prot. Pept. Sci. 16, 182–187 10.2174/1389203716666150120095342 25760347

[75] Al-Ayed M., Alshaybari K., Alshehri D., Jamaan A., Nasser I., Alaamri H. et al. (2019) Obesity and childhood asthma in male schoolchildren in Saudi Arabia: is there a role for leptin, interleukin-4, interleukin-5, and interleukin-21? Ann. Saudi Med. 39, 295–301 10.5144/0256-4947.2019.295 31580718 PMC6832322

[76] Nigro E., Scudiero O., Sarnataro D., Mazzarella G., Sofia M., Bianco A. et al. (2013) Adiponectin affects lung epithelial A549 cell viability counteracting TNFa and IL-1ß toxicity through AdipoR1. Int. J. Biochem. Cell Biol. 45, 1145–1153 10.1016/j.biocel.2013.03.003 23500159

[77] Gulcan E., Bulut I., Toker A. and Gulcan A. (2009) Evaluation of glucose tolerance status in patients with asthma bronchiale. J. Asthma 46, 207–209 10.1080/02770900802627302 19253132

[78] Karampatakis N., Karampatakis T., Galli-Tsinopoulou A., Kotanidou E.P., Tsergouli K., Eboriadou-Petikopoulou M. et al. (2017) Impaired glucose metabolism and bronchial hyperresponsiveness in obese prepubertal asthmatic children. Pediatr. Pulmonol. 52, 160–166 10.1002/ppul.23516 27362543

[79] DeChristopher L.R. and Tucker K.L. (2018) Excess free fructose, high-fructose corn syrup and adult asthma: The Framingham Offspring Cohort. Br. J. Nutr. 119, 1157–1167 10.1017/S0007114518000417 29587887

[80] Sun Q., Li J. and Gao F. (2014) New insights into insulin: the anti-inflammatory effect and its clinical relevance. World J. Diabetes 5, 89, https://pmc/articles/PMC3992527/?report=abstract 10.4239/wjd.v5.i2.89 24765237 PMC3992527

[81] Chang S.C. and Yang W.C.V. (2016) Hyperglycemia, tumorigenesis, and chronic inflammation. Crit. Rev. Oncol. Hematol. 108, 146–153 10.1016/j.critrevonc.2016.11.003 27931833

[82] Youssef-Elabd E.M., McGee K.C., Tripathi G., Aldaghri N., Abdalla M.S., Sharada H.M. et al. (2012) Acute and chronic saturated fatty acid treatment as a key instigator of the TLR-mediated inflammatory response in human adipose tissue, in vitro. J. Nutr. Biochem. 23, 39–50 10.1016/j.jnutbio.2010.11.003 21414768 PMC3243902

[83] Forno E., Han Y.Y., Muzumdar R.H. and Celedón J.C. (2015) Insulin resistance, metabolic syndrome, and lung function in US adolescents with and without asthma. J. Allergy Clin. Immunol. 136, 304.e8–311.e8, https://pmc/articles/PMC4530022/?report=abstract 10.1016/j.jaci.2015.01.010 25748066 PMC4530022

[84] Hill M.J., Metcalfe D. and McTernan P.G. (2009) Obesity and diabetes: lipids, “nowhere to run to”. Clin. Sci. 116, 113–123 10.1042/CS20080050 19076064

[85] Cardoso D. and Perucha E. (2021) Cholesterol metabolism: a new molecular switch to control inflammation. Clin. Sci. 135, 1389–1408 10.1042/CS20201394 PMC8187928 34086048

[86] Mizuta K., Matoba A., Shibata S., Masaki E. and Emala C.W. (2019) Obesity-induced asthma: role of free fatty acid receptors. Japanese Dental Sci. Rev. 55, 103–107 10.1016/j.jdsr.2019.07.002 PMC6728269 31516639

[87] Karpe F., Dickmann J.R. and Frayn K.N. (2011) Fatty acids, obesity, and insulin resistance: time for a reevaluation. Diabetes 60, 2441–2449 10.2337/db11-0425 21948998 PMC3178283

[88] Rogero M.M. and Calder P.C. (2018) Obesity, inflammation, toll-like receptor 4 and fatty acids. Nutrients 10, 432 https://pmc/articles/PMC5946217/?report=abstract 10.3390/nu10040432 PMC5946217 29601492

[89] Wen L., Ley R., Volchkov P., Stranges P., Avanesyan L., Stonebraker A. et al. (2008) Innate immunity and intestinal microbiota in the development of Type 1 diabetes. Nature 455, 1109–1113 10.1038/nature07336 18806780 PMC2574766

[90] Petnicki-Ocwieja T., Hrncir T., Liu Y., Biswas A., Hudcovic T., Tlaskalova-Hogenova H. et al. (2009) Nod2 is required for the regulation of commensal microbiota in the intestine. Proc. Natl. Acad. Sci. U.S.A. 106, 15813–15818 10.1073/pnas.0907722106 19805227 PMC2747201

[91] Harte A., da Silva N., Creely S., McGee K., Billyard T., Youssef-Elabd E. et al. (2010) Elevated endotoxin levels in non-alcoholic fatty liver disease. J. Inflammation 7, 15 10.1186/1476-9255-7-15 20353583 PMC2873499

[92] Sanz Y. and Moya-Pérez A. (2014) Microbiota, inflammation and obesity. Adv. Exp. Med. Biol. 817, 291–317 10.1007/978-1-4939-0897-4_14 24997040

[93] Wood L., Garg M. and Gibson P. (2011) A high-fat challenge increases airway inflammation and impairs bronchodilator recovery in asthma. J. Allergy Clin. Immunol. 127, 1133–1140 10.1016/j.jaci.2011.01.036 21377715

[94] Wood L.G., Li Q., Scott H.A., Rutting S., Berthon B.S., Gibson P.G. et al. (2019) Saturated fatty acids, obesity, and the nucleotide oligomerization domain-like receptor protein 3 (NLRP3) inflammasome in asthmatic patients. J. Allergy Clin. Immunol. 143, 305–315 10.1016/j.jaci.2018.04.037 29857009

[95] Ko S.H., Jeong J., Baeg M.K., do Han K., Kim H.S., Yoon J.S. et al. (2018) Lipid profiles in adolescents with and without asthma: Korea National Health and nutrition examination survey data. Lipids Health Dis. 17, 158 10.1186/s12944-018-0807-4 30021597 PMC6052620

[96] Barochia A.V., Kaler M., Cuento R.A., Gordon E.M., Weir N.A., Sampson M. et al. (2015) Serum Apolipoprotein A-I and large high-density lipoprotein particles are positively correlated with fev1 in atopic asthma. Am. J. Respir. Crit. Care Med. 191, 990–1000 10.1164/rccm.201411-1990OC 25692941 PMC4435459

[97] Scichilone N., Rizzo M., Benfante A., Catania R., Giglio R.V., Nikolic D. et al. (2013) Serum low density lipoprotein subclasses in asthma. Respir. Med. 107, 1866–1872, www.sciencedirect.com 10.1016/j.rmed.2013.09.001 24075885

[98] Scaduto F., Giglio R.V., Benfante A., Nikolic D., Montalto G., Rizzo M. et al. (2018) Serum lipoproteins are not associated with the severity of asthma. Pulm. Pharmacol. Ther. 50, 57–61 10.1016/j.pupt.2018.04.001 29626633

[99] Reyman M., van Houten M.A., van Baarle D., Bosch A.A.T.M., Man W.H., Chu M.L.J.N. et al. (2019) Impact of delivery mode-associated gut microbiota dynamics on health in the first year of life. Nat. Commun. 10, 1–12 31676793 10.1038/s41467-019-13014-7 PMC6825150

[100] David L.A., Maurice C.F., Carmody R.N., Gootenberg D.B., Button J.E., Wolfe B.E. et al. (2014) Diet rapidly and reproducibly alters the human gut microbiome. Nature 505, 559–563 10.1038/nature12820 24336217 PMC3957428

[101] Singh R.K., Chang H.W., Yan D., Lee K.M., Ucmak D., Wong K. et al. (2017) Influence of diet on the gut microbiome and implications for human health. J. Transl. Med. 15, 73, https://translational-medicine.biomedcentral.com/articles/10.1186/s12967-017-1175-y 10.1186/s12967-017-1175-y 28388917 PMC5385025

[102] Odamaki T., Kato K., Sugahara H., Hashikura N., Takahashi S., Xiao J.Z. et al. (2016) Age-related changes in gut microbiota composition from newborn to centenarian: a cross-sectional study. BMC Microbiol. 16, 90 10.1186/s12866-016-0708-5 27220822 PMC4879732

[103] Costello E.K., Lauber C.L., Hamady M., Fierer N., Gordon J.I. and Knight R. (2009) Bacterial community variation in human body habitats across space and time. Science 326, 1694–1697, https://pmc/articles/PMC3602444/?report=abstract 10.1126/science.1177486 19892944 PMC3602444

[104] Leite G.G.S., Weitsman S., Parodi G., Celly S., Sedighi R., Sanchez M. et al. (2020) Mapping the segmental microbiomes in the human small bowel in comparison with stool: A REIMAGINE Study. Dig. Dis. Sci. 1, 3 10.1007/s10620-020-06173-x PMC7419378 32140945

[105] Ley R.E., Bäckhed F., Turnbaugh P., Lozupone C.A., Knight R.D. and Gordon J.I. (2005) Obesity alters gut microbial ecology. Proc. Natl. Acad. Sci. U.S.A. 102, 11070–11075, www.arb-home.de 10.1073/pnas.0504978102 16033867 PMC1176910

[106] Schwiertz A., Taras D., Schäfer K., Beijer S., Bos N.A., Donus C. et al. (2010) Microbiota and SCFA in lean and overweight healthy subjects. Obesity 18, 190–195 10.1038/oby.2009.167 19498350

[107] Carding S., Verbeke K., Vipond D.T., Corfe B.M. and Owen L.J. (2015) Dysbiosis of the gut microbiota in disease. Microbial Ecol. Health Dis. 26, 26191 https://pmc/articles/PMC4315779/ 10.3402/mehd.v26.26191 25651997 PMC4315779

[108] Chiu C.Y., Chan Y.L., Tsai M.H., Wang C.J., Chiang M.H. and Chiu C.C. (2019) Gut microbial dysbiosis is associated with allergen-specific IgE responses in young children with airway allergies. World Allergy Organ. J. 12, 100021 10.1016/j.waojou.2019.100021 30937143 PMC6439417

[109] Lee J.J., Kim S.H., Lee M.J., Kim B.K., Song W.J., Park H.W. et al. (2019) Different upper airway microbiome and their functional genes associated with asthma in young adults and elderly individuals. Allergy 74, 709–719 10.1111/all.13608 30242844

[110] Amabebe E., Robert F.O., Agbalalah T. and Orubu E.S.F. (2020) This is a repository copy of Microbial dysbiosis-induced obesity: role of gut microbiota in homoeostasis of energy metabolism. http://eprints.whiterose.ac.uk/165574/10.1017/S0007114520000380 32008579

[111] Gurung M., Li Z., You H., Rodrigues R., Jump D.B., Morgun A. et al. (2020) Role of gut microbiota in type 2 diabetes pathophysiology. EBioMedicine 51, 102590 10.1016/j.ebiom.2019.11.051 31901868 PMC6948163

[112] Kitai T. and Tang W.H.W. (2018) Gut microbiota in cardiovascular disease and heart failure. Clin. Sci. 132, 85–91 10.1042/CS20171090 PMC6413501 29326279

[113] Harris K.G. and Chang E.B. (2018) The intestinal microbiota in the pathogenesis of inflammatory bowel diseases: New insights into complex disease. Clin. Sci. 132, 2013–2028 10.1042/CS20171110 PMC6907688 30232239

[114] Ghoshal S., Witta J., Zhong J., de Villiers W. and Eckhardt E. (2009) Chylomicrons promote intestinal absorption of lipopolysaccharides. J. Lipid Res. 50, 90–97 10.1194/jlr.M800156-JLR200 18815435

[115] Tulkens J., Vergauwen G., van Deun J., Geeurickx E., Dhondt B., Lippens L. et al. (2020) Increased levels of systemic LPS-positive bacterial extracellular vesicles in patients with intestinal barrier dysfunction. Gut 69, 191–193 10.1136/gutjnl-2018-317726 30518529 PMC6943244

[116] Benard A., Desreumeaux P., Huglo D., Hoorelbeke A., Tonnel A.B. and Wallaert B. (1996) Increased intestinal permeability in bronchial asthma. J. Allergy Clin. Immunol. 97, 1173–1178 10.1016/S0091-6749(96)70181-1 8648009

[117] Hijazi Z., Molla A.M., Al-Habashi H., Muawad W.M.R.A., Mollo A.M. and Sharma P.N. (2004) Intestinal permeability is increased in bronchial asthma. Arch. Dis. Child. 89, 227–229 10.1136/adc.2003.027680 14977697 PMC1719843

[118] Cani P.D., Amar J., Iglesias M.A., Poggi M., Knauf C., Bastelica D. et al. (2007) Metabolic endotoxemia initiates obesity and insulin resistance. Diabetes 56, 1761–1772 10.2337/db06-1491 17456850

[119] Moreira A.P.B., Texeira T.F.S., Ferreira A.B., do Carmo Gouveia Peluzio M. and de Cássia Gonçalves Alfenas R. (2012) Influence of a high-fat diet on gut microbiota, intestinal permeability and metabolic endotoxaemia. Br. J. Nutr. 108, 801–809 10.1017/S0007114512001213 22717075

[120] Walker J., Dieleman L., Mah D., Park K., Meddings J. and Vethanayagam D. (2014) High prevalence of abnormal gastrointestinal permeability in moderate-severe asthma. Clin. Invest. Med. 37, 53–57 10.25011/cim.v37i2.21086 24690419

[121] Genser L., Aguanno D., Soula H.A., Dong L., Trystram L., Assmann K. et al. (2018) Increased jejunal permeability in human obesity is revealed by a lipid challenge and is linked to inflammation and type 2 diabetes. J. Pathol. 246, 217–230 10.1002/path.5134 29984492

[122] Harte A.L., Varma M.C., Tripathi G., Mcgee K.C., Al-Daghri N.M., Al-Attas O.S. et al. (2012) High fat intake leads to acute postprandial exposure to circulating endotoxin in type 2 diabetic subjects. Diabetes Care 35, 375–382 10.2337/dc11-1593 22210577 PMC3263907

[123] Erridge C., Attina T., Spickett C.M. and Webb D.J. (2007) A high-fat meal induces low-grade endotoxemia: evidence of a novel mechanism of postprandial inflammation. Am. J. Clin. Nutr. 86, 1286–1292, https://academic.oup.com/ajcn/article-abstract/86/5/1286/4651083 10.1093/ajcn/86.5.1286 17991637

[124] Lyte J.M., Gabler N.K. and Hollis J.H. (2016) Postprandial serum endotoxin in healthy humans is modulated by dietary fat in a randomized, controlled, cross-over study. Lipids Health Dis. 15, 1–10 10.1186/s12944-016-0357-6 27816052 PMC5097840

[125] Vors C., Pineau G., Drai J., Meugnier E., Pesenti S., Laville M. et al. (2015) Postprandial endotoxemia linked with chylomicrons and lipopolysaccharides handling in obese versus lean men: a Lipid Dose-Effect Trial. J. Clin. Endocrinol. Metab. 100, 3427–3435 10.1210/jc.2015-2518 26151336

[126] Laugerette F., Vors C., Géloën A., Chauvin M.A., Soulage C., Lambert-Porcheron S. et al. (2011) Emulsified lipids increase endotoxemia: possible role in early postprandial low-grade inflammation. J. Nutr. Biochem. 22, 53–59 10.1016/j.jnutbio.2009.11.011 20303729

[127] Creely S.J., McTernan P.G., Kusminski C.M., Fisher F.M., da Silva N.F., Khanolkar M. et al. (2007) Lipopolysaccharide activates an innate immune system response in human adipose tissue in obesity and type 2 diabetes. Am. J. Physiol. Endocrinol. Metab. 292, E740–E747 10.1152/ajpendo.00302.2006 17090751

[128] Guo S., Al-Sadi R., Said H.M. and Ma T.Y. (2013) Lipopolysaccharide causes an increase in intestinal tight junction permeability in vitro and in vivo by inducing enterocyte membrane expression and localization of TLR-4 and CD14. Am. J. Pathol. 182, 375–387 10.1016/j.ajpath.2012.10.014 23201091 PMC3562736

[129] Liu T., Zhang L., Joo D. and Sun S.-C. (2017) NF-κB signaling in inflammation. Signal Transduct. Target. Ther. 2, 1–9, https://www.nature.com/articles/sigtrans201723 10.1038/sigtrans.2017.23 PMC5661633 29158945

[130] Guo S., Nighot M., Al-Sadi R., Alhmoud T., Nighot P. and Ma T.Y. (2015) Lipopolysaccharide regulation of intestinal tight junction permeability is mediated by TLR4 signal transduction pathway activation of FAK and MyD88. J. Immunol. 195, 4999–5010 10.4049/jimmunol.1402598 26466961 PMC4637237

[131] Nighot M., Al-Sadi R., Guo S., Rawat M., Nighot P., Watterson M.D. et al. (2017) Lipopolysaccharide-induced increase in intestinal epithelial tight permeability is mediated by toll-like receptor 4/myeloid differentiation primary response 88 (MyD88) activation of myosin light chain kinase expression. Am. J. Pathol. 187, 2698–2710 10.1016/j.ajpath.2017.08.005 29157665 PMC5718096

[132] Nighot M., Rawat M., Al-Sadi R., Castillo E.F., Nighot P. and Ma T.Y. (2019) Lipopolysaccharide-induced increase in intestinal permeability is mediated by TAK-1 activation of IKK and MLCK/MYLK gene. Am. J. Pathol. 189, 797–812, https://pmc/articles/PMC6446229/?report=abstract 10.1016/j.ajpath.2018.12.016 30711488 PMC6446229

[133] Goleva E., Hauk P.J., Hall C.F., Liu A.H., Riches D.W.H., Martin R.J. et al. (2008) Corticosteroid-resistant asthma is associated with classical antimicrobial activation of airway macrophages. J. Allergy Clin. Immunol. 122, 550, https://pmc/articles/PMC3930345/?report=abstract 10.1016/j.jaci.2008.07.007 18774390 PMC3930345

[134] Goleva E., Jackson L.P., Harris J.K., Robertson C.E., Sutherland E.R., Hall C.F. et al. (2013) The effects of airway microbiome on corticosteroid responsiveness in asthma. Am. J. Respir. Crit. Care Med. 188, 1193–1201 10.1164/rccm.201304-0775OC 24024497 PMC3863730

[135] Hauk P.J., Krawiec M., Murphy J., Boguniewicz J., Schiltz A., Goleva E. et al. (2008) Neutrophilic airway inflammation and association with bacterial lipopolysaccharide in children with asthma and wheezing. Pediatr. Pulmonol. 43, 916–923 10.1002/ppul.20880 18668688

[136] Berger M., de Boer J.D., Bresser P., van der Poll T., Lutter R., Sterk P.J. et al. (2015) Lipopolysaccharide amplifies eosinophilic inflammation after segmental challenge with house dust mite in asthmatics. Allergy 70, 257–264 10.1111/all.12544 25381858

[137] Ren Y., Ichinose T., He M., Youshida S., Nishikawa M. and Sun G. (2019) Co-exposure to lipopolysaccharide and desert dust causes exacerbation of ovalbumin-induced allergic lung inflammation in mice via TLR4/MyD88-dependent and -independent pathways. Allergy Asthma Clin. Immunol. 15, 53–57 10.1186/s13223-019-0396-4 31889961 PMC6921588

[138] Curths C., Wichmann J., Dunker S., Windt H., Hoymann H.G., Lauenstein H.D. et al. (2014) Airway hyper-responsiveness in lipopolysaccharide-challenged common marmosets (callithrix jacchus). Clin. Sci. 126, 155–162 10.1042/CS20130101 PMC3793853 23879175

[139] Blomkalns A.L., Stoll L.L., Shaheen W., Romig-Martin S.A., Dickson E.W., Weintraub N.L. et al. (2011) Low level bacterial endotoxin activates two distinct signaling pathways in human peripheral blood mononuclear cells. J. Inflamm. 8, 4, http://journal-inflammation.biomedcentral.com/articles/10.1186/1476-9255-8-4 10.1186/1476-9255-8-4 PMC3056742 21352551

[140] Hadjigol S., Netto K.G., Maltby S., Tay H.L., Nguyen T.H., Hansbro N.G. et al. (2020) Lipopolysaccharide induces steroid-resistant exacerbations in a mouse model of allergic airway disease collectively through IL-13 and pulmonary macrophage activation. Clin. Exp. Allergy 50, 82–94 10.1111/cea.13505 31579973

[141] Zhao S., Jiang Y., Yang X., Guo D., Wang Y., Wang J. et al. (2017) Lipopolysaccharides promote a shift from Th2-derived airway eosinophilic inflammation to Th17-derived neutrophilic inflammation in an ovalbumin-sensitized murine asthma model. J. Asthma 54, 447–455 10.1080/02770903.2016.1223687 27589490

[142] Jiang Y., Zhao S., Yang X., Liu Y. and Wang C. (2015) Dll4 in the DCs isolated from OVA-sensitized mice is involved in Th17 differentiation inhibition by 1,25-dihydroxyvitamin D3 in vitro. J. Asthma 52, 989–995 10.3109/02770903.2015.1056349 26333305

[143] Yoda Y., Tamura K. and Shima M. (2017) Airborne endotoxin concentrations in indoor and outdoor particulate matter and their predictors in an urban city. Indoor Air 27, 955–964 10.1111/ina.12370 28161889

[144] Farokhi A., Heederik D. and Smit L.A.M. (2018) Respiratory health effects of exposure to low levels of airborne endotoxin - a systematic review. Environ. Health 17, 14, https://ehjournal.biomedcentral.com/articles/10.1186/s12940-018-0360-7 10.1186/s12940-018-0360-7 29422043 PMC5806377

[145] Rolph C., Gwyther C., Tyrrel S., Nasir Z., Drew G., Jackson S. et al. (2018) Sources of airborne endotoxins in ambient air and exposure of nearby communities—a review. Atmosphere 9, 375, http://www.mdpi.com/2073-4433/9/10/375 10.3390/atmos9100375

[146] Barnig C., Reboux G., Roussel S., Casset A., Sohy C., Dalphin J.-C. et al. (2013) Indoor dust and air concentrations of endotoxin in urban and rural environments. Lett. Appl. Microbiol. 56, 161–167 10.1111/lam.12024 23121051

[147] Basinas I., Sigsgaard T., Kromhout H., Heederik D., Wouters I.M. and Schlünssen V. (2015) A comprehensive review of levels and determinants of personal exposure to dust and endotoxin in livestock farming. J. Exposure Sci. Environ. Epidemiol. 25, 123–137, www.nature.com/jes 10.1038/jes.2013.83 24280684

[148] de Rooij M.M.T., Smit L.A.M., Erbrink H.J., Hagenaars T.J., Hoek G., Ogink N.W.M. et al. (2019) Endotoxin and particulate matter emitted by livestock farms and respiratory health effects in neighboring residents. Environ. Int. 132, 105009 10.1016/j.envint.2019.105009 31387023

[149] von Mutius E., Braun-Fahrländer C., Schierl R., Riedler J., Ehlermann S., Maisch S. et al. (2000) Exposure to endotoxin or other bacterial components might protect against the development of atopy. Clin. Exp. Allergy 30, 1230–1234 10.1046/j.1365-2222.2000.00959.x 10971468

[150] Kuipers H., Hijdra D., de Vries V.C., Hammad H., Prins J.-B., Coyle A.J. et al. (2003) Lipopolysaccharide-induced suppression of airway Th2 responses does not require IL-12 production by dendritic cells. J. Immunol. 171, 3645–3654, http://www.jimmunol.org/content/171/7/3645http://www.jimmunol.org/content/171/7/3645.full#ref-list-1 10.4049/jimmunol.171.7.3645 14500662

[151] Zhang X., Zhang X., Zhang N., Wang X., Sun L., Chen N. et al. (2020) Airway microbiome, host immune response and recurrent wheezing in infants with severe respiratory syncytial virus bronchiolitis. Pediatr. Allergy Immunol. 31, 281–289 10.1111/pai.13183 31788862

[152] Thorne P.S., Mendy A., Metwali N., Salo P., Co C., Jaramillo R. et al. (2015) Endotoxin exposure: Predictors and prevalence of associated asthma outcomes in the United States. Am. J. Respir. Crit. Care Med. 192, 1287–1297 10.1164/rccm.201502-0251OC 26258643 PMC4731700

[153] Carnes M.U., Hoppin J.A., Metwali N., Wyss A.B., Hankinson J.L., O’Connell E.L. et al. (2017) House dust endotoxin levels are associated with adult asthma in a U.S. farming population. Ann. Am. Thorac. Soc. 14, 324–331 10.1513/AnnalsATS.201611-861OC 27977294 PMC5427722

[154] Steimle A., Autenrieth I.B. and Frick J.S. (2016) Structure and function: Lipid A modifications in commensals and pathogens. Int. J. Med. Microbiol. 306, 290–301 10.1016/j.ijmm.2016.03.001 27009633

[155] Brix S., Eriksen C., Larsen J.M. and Bisgaard H. (2015) Metagenomic heterogeneity explains dual immune effects of endotoxins. J. Allergy Clin. Immunol. 135, 277–280 10.1016/j.jaci.2014.09.036 25445821

[156] Mendy A., Wilkerson J., Salo P.M., Cohn R.D., Zeldin D.C. and Thorne P.S. (2018) Endotoxin predictors and associated respiratory outcomes differ with climate regions in the U.S HHS Public Access. Environ. Int. 112, 218–226, https://www1.eere.energy.gov/buildings/publications/pdfs/building_america/ 10.1016/j.envint.2017.12.003 29277065 PMC5899028

[157] Lynch S.v., Wood R.A., Boushey H., Bacharier L.B., Bloomberg G.R., Kattan M. et al. (2014) Effects of early-life exposure to allergens and bacteria on recurrent wheeze and atopy in urban children. J. Allergy Clin. Immunol. 134, 593.e12–601.e12 10.1016/j.jaci.2014.04.018 24908147 PMC4151305

[158] Larsen J.M., Musavian H.S., Butt T.M., Ingvorsen C., Thysen A.H. and Brix S. (2015) Chronic obstructive pulmonary disease and asthma-associated Proteobacteria, but not commensal *Prevotella* spp., promote Toll-like receptor 2-independent lung inflammation and pathology. Immunology 144, 333–342 10.1111/imm.12376 25179236 PMC4298427

[159] Barreto M., Evangelisti M., Principessa L., Simmaco M., Negro V., Lionetto L. et al. (2015) Intestinal permeability in children with recurrent respiratory and gastrointestinal symptoms. J. Paediatr. Child Health 51, 1214–1220 10.1111/jpc.12927 26044914

[160] Benson A.K., Kelly S.A., Legge R., Ma F., Low S.J., Kim J. et al. (2010) Individuality in gut microbiota composition is a complex polygenic trait shaped by multiple environmental and host genetic factors. Proc. Natl. Acad. Sci. U.S.A. 107, 18933–18938, https://www.pnas.org/content/107/44/18933 10.1073/pnas.1007028107 20937875 PMC2973891

[161] Verdam F., Fuentes S., de Jonge C., Zoetendal E., Erbil R., Greve J. et al. (2013) Human intestinal microbiota composition is associated with local and systemic inflammation in obesity. Obesity 21, 607–615 10.1002/oby.20466 23526699

[162] Huang Y.J., Nelson C.E., Brodie E.L., Desantis T.Z., Baek M.S., Liu J. et al. (2011) Airway microbiota and bronchial hyperresponsiveness in patients with suboptimally controlled asthma. J. Allergy Clin. Immunol. 127, 372–381 10.1016/j.jaci.2010.10.048 21194740 PMC3037020

[163] Taylor S.L., Leong L.E.X., Choo J.M., Wesselingh S., Yang I.A., Upham J.W. et al. (2018) Inflammatory phenotypes in patients with severe asthma are associated with distinct airway microbiology. J. Allergy Clin. Immunol. 141, 94.e15–103.e15 10.1016/j.jaci.2017.03.044 28479329

[164] Pang Z., Wang G., Gibson P., Guan X., Zhang W., Zheng R. et al. (2019) Airway microbiome in different inflammatory phenotypes of asthma: a cross-sectional study in northeast China. Int. J. Med. Sci. 16, 477–485 10.7150/ijms.29433 30911282 PMC6428974

[165] Zhang Q., Cox M., Liang Z., Brinkmann F., Cardenas P.A., Duff R. et al. (2016) Airway microbiota in severe asthma and relationship to asthma severity and phenotypes. PLoS ONE 11, e0152724 10.1371/journal.pone.0152724 27078029 PMC4831690

[166] Depner M., Ege M.J., Cox M.J., Dwyer S., Walker A.W., Birzele L.T. et al. (2017) Bacterial microbiota of the upper respiratory tract and childhood asthma. J. Allergy Clin. Immunol. 139, 826.e13–834.e13 10.1016/j.jaci.2016.05.050 27576124

[167] Yang X., Li H., Ma Q., Zhang Q. and Wang C. (2018) Neutrophilic asthma is associated with increased airway bacterial burden and disordered community composition. Biomed Res. Int. 2018, 9230234 10.1155/2018/9230234 30105264 PMC6076954

[168] Zhou Y., Jackson D., Bacharier L.B., Mauger D., Boushey H., Castro M. et al. (2019) The upper-airway microbiota and loss of asthma control among asthmatic children. Nat. Commun. 10, 5714 10.1038/s41467-019-13698-x 31844063 PMC6915697

[169] Fazlollahi M., Lee T.D., Andrade J., Oguntuyo K., Chun Y., Grishina G. et al. (2018) The nasal microbiome in asthma. J. Allergy Clin. Immunol. 142, 834.e2–843.e2 10.1016/j.jaci.2018.02.020 29518419 PMC6123291

[170] Huang Y.J., Nariya S., Harris J.M., Lynch S.v., Choy D.F., Arron J.R. et al. (2015) The airway microbiome in patients with severe asthma: associations with disease features and severity. J. Allergy Clin. Immunol. 136, 874–884 10.1016/j.jaci.2015.05.044 26220531 PMC4600429

[171] Turnbaugh P.J., Ley R.E., Mahowald M.A., Magrini V., Mardis E.R. and Gordon J.I. (2006) An obesity-associated gut microbiome with increased capacity for energy harvest. Nature 444, 1027–1031 10.1038/nature05414 17183312

[172] Bäckhed F., Ding H., Wang T., Hooper L.v., Gou Y.K., Nagy A. et al. (2004) The gut microbiota as an environmental factor that regulates fat storage. Proc. Natl. Acad. Sci. U.S.A. 101, 15718–15723 10.1073/pnas.0407076101 15505215 PMC524219

[173] Bäckhed F., Manchester J.K., Semenkovich C.F. and Gordon J.I. (2007) Mechanisms underlying the resistance to diet-induced obesity in germ-free mice. Proc. Natl. Acad. Sci. U.S.A. 104, 979–984 10.1073/pnas.0605374104 17210919 PMC1764762

[174] Anhê F.F., Jensen B.A.H., Varin T.v., Servant F., van Blerk S., Richard D. et al. (2020) Type 2 diabetes influences bacterial tissue compartmentalisation in human obesity. Nat. Metab. 2, 233–242 10.1038/s42255-020-0178-9 32694777

[175] Marsland B.J., Trompette A. and Gollwitzer E.S. (2015) The gut-lung axis in respiratory disease. Annals of the American Thoracic Society, pp. S150–S156, American Thoracic Society 10.1513/AnnalsATS.201503-133AW 26595731

[176] Abrahamsson T.R., Jakobsson H.E., Andersson A.F., Björkstén B., Engstrand L. and Jenmalm M.C. (2014) Low gut microbiota diversity in early infancy precedes asthma at school age. Clin. Exp. Allergy 44, 842–850 10.1111/cea.12253 24330256

[177] Arrieta M.C., Stiemsma L.T., Dimitriu P.A., Thorson L., Russell S., Yurist-Doutsch S. et al. (2015) Early infancy microbial and metabolic alterations affect risk of childhood asthma. Sci. Transl. Med. 7, 307ra152 10.1126/scitranslmed.aab2271 26424567

[178] Arrieta M.C., Arévalo A., Stiemsma L., Dimitriu P., Chico M.E., Loor S. et al. (2018) Associations between infant fungal and bacterial dysbiosis and childhood atopic wheeze in a nonindustrialized setting. J. Allergy Clin. Immunol. 142, 424.e10–434.e10 10.1016/j.jaci.2017.08.041 29241587 PMC6075469

[179] Fujimura K.E., Sitarik A.R., Havstad S., Lin D.L., Levan S., Fadrosh D. et al. (2016) Neonatal gut microbiota associates with childhood multisensitized atopy and T cell differentiation. Nat. Med. 22, 1187–1191 10.1038/nm.4176 27618652 PMC5053876

[180] Stokholm J., Blaser M.J., Thorsen J., Rasmussen M.A., Waage J., Vinding R.K. et al. (2018) Maturation of the gut microbiome and risk of asthma in childhood. Nat. Commun. 9, 141 10.1038/s41467-017-02573-2 29321519 PMC5762761

[181] Stiemsma L.T., Arrieta M.C., Dimitriu P.A., Cheng J., Thorson L., Lefebvre D.L. et al. (2016) Shifts in Lachnospira and Clostridium sp. in the 3-month stool microbiome are associated with preschool age asthma. Clin. Sci. 130, 2199–2207 10.1042/CS20160349 27634868

[182] Zhang Y., Li T., Yuan H., Pan W. and Dai Q. (2018) Correlations of inflammatory factors with intestinal flora and gastrointestinal incommensurate symptoms in children with asthma. Med. Sci. Monit. 24, 7975–7979 10.12659/MSM.910854 30401793 PMC6234755

[183] Metsälä J., Lundqvist A., Virta L.J., Kaila M., Gissler M. and Virtanen S.M. (2015) Prenatal and post-natal exposure to antibiotics and risk of asthma in childhood. Clin. Exp. Allergy 45, 137–145 10.1111/cea.12356 24943808

[184] Ni J., Friedman H., Boyd B.C., McGurn A., Babinski P., Markossian T. et al. (2019) Early antibiotic exposure and development of asthma and allergic rhinitis in childhood. BMC Pediatr. 19, 225 10.1186/s12887-019-1594-4 31277618 PMC6612173

[185] Begley L., Madapoosi S., Opron K., Ndum O., Baptist A., Rysso K. et al. (2018) Gut microbiota relationships to lung function and adult asthma phenotype: a pilot study. BMJ Open Respir. Res. 5, 1–7 10.1136/bmjresp-2018-000324 PMC6157510 30271607

[186] Qian L.J., Kang S.M., Xie J.L., Huang L., Wen Q., Fan Y.Y. et al. (2017) Early-life gut microbial colonization shapes Th1/Th2 balance in asthma model in BALB/c mice. BMC Microbiol. 17, 1–8 10.1186/s12866-017-1044-0 28623898 PMC5473985

[187] Russell S.L., Gold M.J., Hartmann M., Willing B.P., Thorson L., Wlodarska M. et al. (2012) Early life antibiotic-driven changes in microbiota enhance susceptibility to allergic asthma. EMBO Rep. 13, 440–447 10.1038/embor.2012.32 22422004 PMC3343350

[188] Cait A., Hughes M.R., Antignano F., Cait J., Dimitriu P.A., Maas K.R. et al. (2018) Microbiome-driven allergic lung inflammation is ameliorated by short-chain fatty acids. Mucosal Immunol. 11, 785–795 10.1038/mi.2017.75 29067994

[189] Alhasan M.M., Cait A.M., Heimesaat M.M., Blaut M., Klopfleisch R., Wedel A. et al. (2020) Antibiotic use during pregnancy increases offspring asthma severity in a dose-dependent manner. Allergy 75, 1–12 10.1111/all.14234 32064643

[190] Hill C., Guarner F., Reid G., Gibson G.R., Merenstein D.J., Pot B. et al. (2014) Expert consensus document: The International Scientific Association for Probiotics and Prebiotics consensus statement on the scope and appropriate use of the term probiotic. Nat. Rev. Gastroenterol. Hepatol. 11, 506–514 10.1038/nrgastro.2014.66 24912386

[191] Gibson G.R., Hutkins R., Sanders M.E., Prescott S.L., Reimer R.A., Salminen S.J. et al. (2017) Expert consensus document: The International Scientific Association for Probiotics and Prebiotics (ISAPP) consensus statement on the definition and scope of prebiotics. Nat. Rev. Gastroenterol. Hepatol. 14, 491–502 10.1038/nrgastro.2017.75 28611480

[192] Klemashevich C., Wu C., Howsmon D., Alaniz R.C., Lee K. and Jayaraman A. (2014) Rational identification of diet-derived postbiotics for improving intestinal microbiota function. Curr. Opin. Biotechnol. 26, 85–90 10.1016/j.copbio.2013.10.006 24679263

[193] Hernández-Granados M.J. and Franco-Robles E. (2020) Postbiotics in human health: possible new functional ingredients? Food Res. Int. 137, 109660 10.1016/j.foodres.2020.109660 33233239

[194] Gill P.A., van Zelm M.C., Muir J.G. and Gibson P.R. (2018) Review article: short chain fatty acids as potential therapeutic agents in human gastrointestinal and inflammatory disorders. Aliment. Pharmacol. Ther. 48, 15–34, https://onlinelibrary.wiley.com/doi/full/10.1111/apt.14689 10.1111/apt.14689 29722430

[195] Holmes Z.C., Silverman J.D., Dressman H.K., Wei Z., Dallow E.P., Armstrong S.C. et al. (2020) Short-chain fatty acid production by gut microbiota from children with obesity differs according to prebiotic choice and bacterial community composition. mBio 11, e00914–20 https://mbio.asm.org/content/11/4/e00914-20 10.1128/mBio.00914-20 32788375 PMC7439474

[196] Liu T., Li J., Liu Y., Xiao N., Suo H., Xie K. et al. (2012) Short-chain fatty acids suppress lipopolysaccharide-Induced production of nitric oxide and proinflammatory cytokines through inhibition of NF-κB Pathway in RAW264.7 cells. Inflammation 35, 1676–1684 10.1007/s10753-012-9484-z 22669487

[197] Wang F., Liu J., Weng T., Shen K., Chen Z., Yu Y. et al. (2017) The inflammation induced by lipopolysaccharide can be mitigated by short-chain fatty acid, butyrate, through upregulation of IL-10 in septic shock. Scand. J. Immunol. 85, 258–263 10.1111/sji.12515 27943364

[198] Theiler A., Bärnthaler T., Platzer W., Richtig G., Peinhaupt M., Rittchen S. et al. (2019) Butyrate ameliorates allergic airway inflammation by limiting eosinophil trafficking and survival. J. Allergy Clin. Immunol. 144, 764–776 10.1016/j.jaci.2019.05.002 31082458

[199] Pluznick J.L., Protzko R.J., Gevorgyan H., Peterlin Z., Sipos A., Han J. et al. (2013) Olfactory receptor responding to gut microbiotaderived signals plays a role in renin secretion and blood pressure regulation. Proc. Natl. Acad. Sci. U.S.A. 110, 4410–4415 10.1073/pnas.1215927110 23401498 PMC3600440

[200] Li M., van Esch B.C.A.M., Henricks P.A.J., Folkerts G. and Garssen J. (2018) The anti-inflammatory effects of short chain fatty acids on lipopolysaccharide- or tumor necrosis factor α-stimulated endothelial cells via activation of GPR41/43 and inhibition of HDACs. Front. Pharmacol. 9, 533, https://www.frontiersin.org/article/10.3389/fphar.2018.00533/full 10.3389/fphar.2018.00533 29875665 PMC5974203

[201] Halnes I., Baines K.J., Berthon B.S., MacDonald-Wicks L.K., Gibson P.G. and Wood L.G. (2017) Soluble fibre meal challenge reduces airway inflammation and expression of GPR43 and GPR41 in asthma. Nutrients 9, 57 www.mdpi.com/journal/nutrients 10.3390/nu9010057 28075383 PMC5295101

[202] Mizuta K., Sasaki H., Zhang Y., Matoba A. and Emala C.W. (2020) The short-chain free fatty acid receptor FFAR3 is expressed and potentiates contraction in human airway smooth muscle. Am. J. Physiol. Lung Cell. Mol. Physiol. 318, L1248–L1260 10.1152/ajplung.00357.2019 32209026 PMC7347267

[203] Ivashkin V., Zolnikova O., Potskherashvili N., Trukhmanov A., Kokina N., Sedova A. et al. (2019) Metabolic activity of intestinal microflora in patients with bronchial asthma. Clin. Pract. 9, 1–5 10.4081/cp.2019.1126 PMC6401556 30931087

[204] Lee-Sarwar K.A., Lasky-Su J., Kelly R.S., Litonjua A.A. and Weiss S.T. (2020) Gut microbial-derived metabolomics of asthma. Metabolites 10, 97 10.3390/metabo10030097 PMC7142494 32155960

[205] Peng L., Li Z.-R., Green R.S., Holzman I.R. and Lin J. (2009) Butyrate enhances the intestinal barrier by facilitating tight junction assembly via activation of AMP-activated protein kinase in Caco-2 cell monolayers. J. Nutr. 139, 1619–1625 10.3945/jn.109.104638 19625695 PMC2728689

[206] Wang H.B., Wang P.Y., Wang X., Wan Y.L. and Liu Y.C. (2012) Butyrate enhances intestinal epithelial barrier function via up-regulation of tight junction protein claudin-1 transcription. Dig. Dis. Sci. 57, 3126–3135 10.1007/s10620-012-2259-4 22684624

[207] Yan H. and Ajuwon K.M. (2017) Butyrate modifies intestinal barrier function in IPEC-J2 cells through a selective upregulation of tight junction proteins and activation of the Akt signaling pathway. PLoS ONE 12, e0179586 10.1371/journal.pone.0179586 28654658 PMC5487041

[208] Feng Y., Wang Y., Wang P., Huang Y. and Wang F. (2018) Short-chain fatty acids manifest stimulative and protective effects on intestinal barrier function through the inhibition of NLRP3 inflammasome and autophagy. Cell. Physiol. Biochem. 49, 190–205, https://www.karger.com/Article/FullText/492853 10.1159/000492853 30138914

[209] Nielsen D.S.G., Jensen B.B., Theil P.K., Nielsen T.S., Knudsen K.E.B. and Purup S. (2018) Effect of butyrate and fermentation products on epithelial integrity in a mucus-secreting human colon cell line. J. Funct. Foods 40, 9–17 10.1016/j.jff.2017.10.023

[210] Diao H., Jiao A.R., Yu B., Mao X.B. and Chen D.W. (2019) Gastric infusion of short-chain fatty acids can improve intestinal barrier function in weaned piglets. Genes Nutr. 14, 1–16 10.1186/s12263-019-0626-x 30761185 PMC6359775

[211] Williams N.C., Johnson M.A., Shaw D.E., Spendlove I., Vulevic J., Sharpe G.R. et al. (2016) A prebiotic galactooligosaccharide mixture reduces severity of hyperpnoea-induced bronchoconstriction and markers of airway inflammation. Br. J. Nutr. 116, 798–804 10.1017/S0007114516002762 27523186

[212] McLoughlin R., Berthon B.S., Rogers G.B., Baines K.J., Leong L.E.X., Gibson P.G. et al. (2019) Soluble fibre supplementation with and without a probiotic in adults with asthma: a 7-day randomised, double blind, three way cross-over trial. EBioMedicine 46, 473–485 10.1016/j.ebiom.2019.07.048 31375426 PMC6712277

[213] Verheijden K.A.T., Akbari P., Willemsen L.E.M., Kraneveld A.D., Folkerts G., Garssen J. et al. (2015) Inflammation-induced expression of the alarmin interleukin 33 can be suppressed by galacto-oligosaccharides. Int. Arch. Allergy Immunol. 167, 127–136 10.1159/000437327 26304032

[214] Verheijden K.A.T., Willemsen L.E.M., Braber S., Leusink-Muis T., Delsing D.J.M., Garssen J. et al. (2015) Dietary galacto-oligosaccharides prevent airway eosinophilia and hyperresponsiveness in a murine house dust mite-induced asthma model. Respir. Res. 16, 1–9 10.1186/s12931-015-0171-0 25849971 PMC4327967

[215] Verheijden K.A.T., Braber S., Leusink-Muis T., Jeurink P.v., Thijssen S., Kraneveld A.D. et al. (2018) The combination therapy of dietary galacto-oligosaccharides with budesonide reduces pulmonary Th2 driving mediators and mast cell degranulation in a murine model of house dust mite induced asthma. Front. Immunol. 9, 2419 10.3389/fimmu.2018.02419 30405619 PMC6207001

[216] Sabico S., Al-Mashharawi A., Al-Daghri N.M., Wani K., Amer O.E., Hussain D.S. et al. (2019) Effects of a 6-month multi-strain probiotics supplementation in endotoxemic, inflammatory and cardiometabolic status of T2DM patients: a randomized, double-blind, placebo-controlled trial. Clin. Nutr. 38, 1561–1569 10.1016/j.clnu.2018.08.009 30170781

[217] Burton K.J., Rosikiewicz M., Pimentel G., Bütikofer U., von Ah U., Voirol M.J. et al. (2017) Probiotic yogurt and acidified milk similarly reduce postprandial inflammation and both alter the gut microbiota of healthy, young men. Br. J. Nutr. 117, 1312–1322 10.1017/S0007114517000885 28558854

[218] Leber B., Tripolt N.J., Blattl D., Eder M., Wascher T.C., Pieber T.R. et al. (2012) The influence of probiotic supplementation on gut permeability in patients with metabolic syndrome: an open label, randomized pilot study. Eur. J. Clin. Nutr. 66, 1110–1115 10.1038/ejcn.2012.103 22872030

[219] Sabico S., Al-Mashharawi A., Al-Daghri N.M., Yakout S., Alnaami A.M., Alokail M.S. et al. (2017) Effects of a multi-strain probiotic supplement for 12 weeks in circulating endotoxin levels and cardiometabolic profiles of medication naïve T2DM patients: a randomized clinical trial. J. Transl. Med. 15, 249, https://translational-medicine.biomedcentral.com/articles/10.1186/s12967-017-1354-x 10.1186/s12967-017-1354-x 29228964 PMC5725828

[220] Kalliomäki M., Salminen S., Arvilommi H., Kero P., Koskinen P. and Isolauri E. (2001) Probiotics in primary prevention of atopic disease: a randomised placebo-controlled trial. Lancet 357, 1076–1079 10.1016/S0140-6736(00)04259-8 11297958

[221] Kalliomäki M., Salminen S., Poussa T., Arvilommi H. and Isolauri E. (2003) Probiotics and prevention of atopic disease: 4-year follow-up of a randomised placebo-controlled trial. Lancet 361, 1869–1871 10.1016/S0140-6736(03)13490-3 12788576

[222] Kalliomäki M., Salminen S., Poussa T. and Isolauri E. (2007) Probiotics during the first 7 years of life: a cumulative risk reduction of eczema in a randomized, placebo-controlled trial. J. Allergy Clin. Immunol. 119, 1019–1021 10.1016/j.jaci.2006.12.608 17289135

[223] Durack J., Kimes N.E., Lin D.L., Rauch M., McKean M., McCauley K. et al. (2018) Delayed gut microbiota development in high-risk for asthma infants is temporarily modifiable by Lactobacillus supplementation. Nat. Commun. 9, 1–9 10.1038/s41467-018-03157-4 29453431 PMC5816017

[224] Cabana M.D., McKean M., Caughey A.B., Fong L., Lynch S., Wong A. et al. (2017) Early probiotic supplementation for eczema and asthma prevention: a randomized controlled trial. Pediatrics 140, e20163000 10.1542/peds.2016-3000 28784701 PMC5574725

[225] Bergmann K.R., Liu S.X.L., Tian R., Kushnir A., Turner J.R., Li H.L. et al. (2013) Bifidobacteria stabilize claudins at tight junctions and prevent intestinal barrier dysfunction in mouse necrotizing enterocolitis. Am. J. Pathol. 182, 1595–1606 10.1016/j.ajpath.2013.01.013 23470164 PMC3644725

[226] Ling X., Linglong P., Weixia D. and Hong W. (2016) Protective effects of bifidobacterium on intestinal barrier function in LPS-induced enterocyte barrier injury of Caco-2 monolayers and in a rat NEC model. PLoS ONE 11, 10.1371/journal.pone.0161635 PMC4995054 27551722

[227] Ahmadi S., Wang S., Nagpal R., Wang B., Jain S., Razazan A. et al. (2020) A human-origin probiotic cocktail ameliorates aging-related leaky gut and inflammation via modulating the microbiota/taurine/tight junction axis. JCI Insight 5, e132055 https://pmc/articles/PMC7253024/?report=abstract 10.1172/jci.insight.132055 32302292 PMC7253024

[228] Sagar S., Vos A.P., Morgan M.E., Garssen J., Georgiou N.A., Boon L. et al. (2014) The combination of Bifidobacterium breve with non-digestible oligosaccharides suppresses airway inflammation in a murine model for chronic asthma. Biochim. Biophys. Acta Mol. Basis Dis. 1842, 573–583 10.1016/j.bbadis.2014.01.005 24440361

[229] Krumbeck J.A., Maldonado-Gomez M.X., Martínez I., Frese S.A., Burkey T.E., Rasineni K. et al. (2015) In vivo selection to identify bacterial strains with enhanced ecological performance in synbiotic applications. Appl. Environ. Microbiol. 81, 2455–2465 10.1128/AEM.03903-14 25616794 PMC4357922

[230] Krumbeck J.A., Rasmussen H.E., Hutkins R.W., Clarke J., Shawron K., Keshavarzian A. et al. (2018) Probiotic Bifidobacterium strains and galactooligosaccharides improve intestinal barrier function in obese adults but show no synergism when used together as synbiotics. Microbiome 6, 121 10.1186/s40168-018-0494-4 29954454 PMC6022452

[231] Roberts J.D., Suckling C.A., Peedle G.Y., Murphy J.A., Dawkins T.G. and Roberts M.G. (2016) An exploratory investigation of endotoxin levels in novice long distance triathletes, and the effects of a multi-strain probiotic/prebiotic, antioxidant intervention. Nutrients 8, 733 https://pmc/articles/PMC5133117/ 10.3390/nu8110733 PMC5133117 27869661

[232] Haghighat N., Mohammadshahi M., Shayanpour S. and Haghighizadeh M.H. (2019) Effects of synbiotics and probiotics supplementation on serum levels of endotoxin, heat shock protein 70 antibodies and inflammatory markers in hemodialysis patients: a randomized double-blinded controlled trial. Probiotics Antimicrob. Proteins 12, 144–151, https://link.springer.com/article/10.1007/s12602-018-9509-5 10.1007/s12602-018-9509-5 30617950

[233] Aliasgharzadeh A., Dehghan P., Gargari B. and Asghari-Jafarabadi M. (2015) Resistant dextrin, as a prebiotic, improves insulin resistance and inflammation in women with type 2 diabetes: a randomised controlled clinical trial. Br. J. Nutr. 113, 321–330 10.1017/S0007114514003675 27028002

[234] Dehghan P., Gargari B., Jafar-Abadi M. and Aliasgharzadeh A. (2014) Inulin controls inflammation and metabolic endotoxemia in women with type 2 diabetes mellitus: a randomized-controlled clinical trial. Int. J. Food Sci. Nutr. 65, 117–123 10.3109/09637486.2013.836738 24059649

[235] Dehghan P., Pourghassem Gargari B. and Asghari Jafar-abadi M. (2014) Oligofructose-enriched inulin improves some inflammatory markers and metabolic endotoxemia in women with type 2 diabetes mellitus: a randomized controlled clinical trial. Nutrition 30, 418–423 10.1016/j.nut.2013.09.005 24332524

[236] Parnell J., Klancic T. and Reimer R. (2017) Oligofructose decreases serum lipopolysaccharide and plasminogen activator inhibitor-1 in adults with overweight/obesity. Obesity 25, 510–513 10.1002/oby.21763 28229548

[237] Xiao S., Fei N., Pang X., Shen J., Wang L., Zhang B. et al. (2014) A gut microbiota-targeted dietary intervention for amelioration of chronic inflammation underlying metabolic syndrome. FEMS Microbiol. Ecol. 87, 357–367 10.1111/1574-6941.12228 24117923 PMC4255291

